# Eight new species and an annotated checklist of Microgastrinae (Hymenoptera, Braconidae) from Canada and Alaska

**DOI:** 10.3897/zookeys.63.565

**Published:** 2010-10-19

**Authors:** Jose L. Fernández-Triana

**Affiliations:** University of Guelph, Biodiversity Institute of Ontario, Guelph, Ontario, N1G 2W1

**Keywords:** Microgastrinae, Canada, Alaska, new species, checklist, DNA barcoding, diversity

## Abstract

Based on the study of 12,000+ specimens, an annotated checklist of 28 genera and 225 species of Microgastrinae braconids from Canada and Alaska is provided, increasing by 50% the number of species for the region. The genera Distatrix, Iconella, Protomicroplitis and Pseudapanteles for Canada, and Diolcogaster for Alaska are recorded for the first time; all but Iconella and Protomicroplitis represent the northernmost extension of their known distribution. Eight new species are described: Apanteles huberi **sp. n.**, Apanteles jenniferae **sp. n.**, Apanteles masmithi **sp. n.**, Apanteles roughleyi **sp. n.**, Apanteles samarshalli **sp. n.**, Distatrix carolinae **sp. n.**, Pseudapanteles gouleti **sp. n.**, and Venanus heberti **sp. n.** For the more diverse genera, especially Cotesia, Microplitis, Apanteles, Dolichogenidea and Glyptapanteles, many more species are expected to be found. DNA barcode sequences (cytochrome c oxidase I, or CO1) for 3,500+ specimens provided an additional layer of useful data. CO1 sequences were incorporated to the new species descriptions whenever possible, helped to clarify the limits of some species, and flagged cases where further study is needed. Preliminary results on the latitudinal gradient of species/genera richness (45–80° N); as well as biogeographical affinities of the Canadian/Alaska fauna, are discussed. Taking into account the number of specimens in collections still to be studied, data from the barcoded specimens, and extrapolations from Lepidoptera diversity (the host group of the subfamily) the actual diversity of Microgastrinae in the region is estimated to be at least twice that currently known.

## Introduction

Microgastrinae are the single most important group of Lepidoptera parasitoids (Whitfield 1995, 1997), and with over 2,000 described species, rank as the second most diverse subfamily of Braconidae (Yu et al. 2005; Jones et al. 2009). The actual diversity of the group has been estimated at 4,000–10,000 species worldwide (e.g. Mason 1981; Dolphin and Quicke 2001; Jones et al. 2009).

The Catalogue of Nearctic Hymenoptera (Marsh 1979) recorded 124 species of Microgastrinae in Canada and Alaska, a number that 30 years later had increased to 150 (data compiled after Yu et al. 2005; Fernández-Triana et al. 2009b). However, those numbers represent just a fraction of the actual diversity of the group, a fact that has become more evident recently with the examination of extensive material collected throughout the region and the advent of new techniques (such as DNA barcoding) that have been made available for the study of the subfamily.

In this paper eight new species are described; and an updated checklist of the Canadian and Alaskan Microgastrinae is provided with known distribution, taxonomic and/or biological comments when necessary.

## Methods

This study is based mostly on the study of the Microgastrinae housed in the Canadian National Collection of Insects (CNC). CNC is one of the largest collections of the group in the world with over 100,000 pinned specimens plus many thousands more in alcohol (Fernández 2007). The scope of the CNC is worldwide but the strongest representation is from the Nearctic, especially Canada. More than 11,000 Canadian specimens and around 1,000 from Alaska were reviewed, but a significant amount of material still awaits study.

Other collections (curator names provided between brackets), were partially studied and their data were used to compile the distribution of species by provinces.:

–	Great Lakes Forestry Centre, Sault Ste Marie, ON [Kevin Barber, Kathryn Nystrom]. A few hundred specimens reared from Choristoneura spp. (Tortricidae), and from Lepidoptera on blueberry. Geographical scope: mostly ON.

–	J. B. Wallis Museum, University of Manitoba, Winnipeg, MB [Rob Roughley]. A few dozen specimens. Geographical scope: MB and SK.

–	Laurentian Forestry Centre, Ste.-Foy, QC [Jan Klimaszewski, Karine Savard]. A few hundred specimens, many of them reared. Geographical scope: QC.

–	Lyman Museum, McGill University, Montreal, QC [Stephanie Boucher]. Around 400 specimens. Geographical scope: Canada.

–	Northern Forestry Centre, Edmonton, AB [David Langor, Daryl Williams]. A few hundred specimens, many of them reared. Geographical scope: AB, NL.

–	University of Guelph Insect Collection, Guelph, ON [Steve Marshall]. A few hundred specimens. Geographical scope: ON.

–	University of Fairbanks, AK [Derek Sikes, Matthew Bowser]. All Microgastrinae (few dozen specimens). Geographical scope: AK.

–	University of Toronto, Faculty of Forestry, Toronto, ON [Sandy Smith, Laura Timms, Nurul Islam]. Around 400 specimens were studied. Geographical scope: ON.

–	Pacific Forestry Centre, Victoria, BC [Imre Otvos]. Several thousand air-dried specimens in gelatin capsules, reared from Choristoneura spp. were checked with one hundred randomly selected and mounted for further study. Geographical scope: BC.

Whitfield (1995) provided a much needed updated list of the Nearctic Microgastrinae, and assigned to genus all species from the region not treated by Mason (1981). Van Achterberg (2002b) proposed a radical reduction in the number of Microgastrinae genera, and re-arranged all western Palearctic species accordingly (some of those species are also found in Canada and/or Alaska). His modifications were incorporated in the Ichneumonoidea section of Taxapad (Yu et al. 2005), and also the Fauna Europaea website (van Achterberg 2004). Although certainly valid in some regards, van Achterberg (2002b) was based mostly on Holarctic species and contains some decisions not fully supported by additional data (as stated in his paper, more details were intended to be provided later, but there are no published developments to date). Until a study of the microgastrine fauna at world level is available, it seems premature to adopt van Achterberg’s classification (Broad et al. 2009). Therefore, here I am following Whitfield (1995) as the standard for generic and species limits for the Nearctic. The only exceptions are: Dolichogenidea breviventris (Ratzeburg, 1848), where I am following Papp (1978); and Glyptapanteles pallipes (Reinhard, 1880), where I am following Papp (1983). Those two cases are further explained in the annotated checklist.

The new species described in this paper are of importance in biological control efforts (3 species of Apanteles (Fernández-Triana and Huber 2010)), represent the northernmost record of two genera (one species each of Distatrix and Pseudapanteles), are bizarre species (one Apanteles) or illustrate the potential of integrating barcoding with traditional taxonomy (Venanus and one species of Apanteles). Morphologial terms follow those of Huber and Sharkey (1993), and Sharkey and Wharton (1997), with some additional measurements following Mason (1981) and Valerio et al. (2009). When providing measurements, the first figure is that of the holotype, followed by the range for the rest of the specimens if different. For the holotypes a detailed transcription of all labels is provided. All types are deposited in the CNC.

Whenever possible, DNA barcoding (henceforth referred as “barcoding”) data for the new species were added to the descriptions. DNA extraction, PCR and sequencing were done at the Canadian Centre for DNA Barcoding (University of Guelph, ON). DNA extracts were prepared from small pieces of legs using a glass fibre protocol. Extracts were resuspended in 30 μl of dH_2_O, and a 658-bp region near the 5’ terminus of the COI gene was amplified using primers (LepF1–LepR1) following standard protocols (Ivanova et al. 2006). Composite sequences or CO1 fragments smaller than the barcode standard were generated using internal primers when initial amplification was not successful. Sequence divergences were calculated using the K2P distance model (Kimura 1980) and a NJ tree of distances was generated using the MEGA software (Tamura et al. 2007) to provide a graphic representation of the species divergences. Full details of methodology are as in Smith et al. (2008).

For barcoded specimens, the Supplementary Appendices 1–3 show their Sample ID and Process ID from BOLD (Barcoding of Life Data systems, www.barcodinglife.org). Sample IDs allow retrieval of all information associated with a particular specimen from the BOLD database, while Process IDs provide information about the sequence, trace files, laboratory processing, etc. Genbank accession numbers for the type material correspond to records HQ200902-HQ200929.

All genera, and species within each genus, are ordered alphabetically in the annotated checklist. General comments about species diversity, both reported here and estimated, availability of taxonomical reviews, and specimens in collections are provided for every genus. A detailed distribution within Canadian provinces and territories is provided for every species; acronyms follow the Canada Post standard (http://www.canadapost.ca/tools/pg/manual/PGaddress-e.asp).

Distribution outside of Alaska/Canada, based on data from Yu et al. (2005), is also briefly mentioned, using the following acronyms: ENA, CNA and WNA (eastern, central and western North America), PAL (Palearctic), HOL (Holarctic), and NEO (Neotropical).

Biological information is provided only when new or relevant. No intent has been made here to comprehensively deal with the hosts of Microgastrinae in the region. More than 10,000 reared but unidentified specimens in the CNC are currently under study; those results, when available, will be published elsewhere.

It was not possible to establish the specific identity of 29 species (13%) with certainty. They are recorded here only to genus followed by a number (e.g. Cotesia sp. 1) and information on the specimens examined is provided. In most cases, study of the Holarctic fauna will be needed before determining their status.

## Results and discussion

A total of 28 genera and at least 225 species are recorded for Canada and Alaska, representing a 50% increase in the number of known species (Table 1). The genera Distatrix, Iconella, Protomicroplitis and Pseudapanteles for Canada, and Diolcogaster for Alaska are recorded for the first time. Except for Iconella and Protomicroplitis, these records also represent the northernmost extension of their known distribution.

**Table 1.** Number of species of Microgastrinae in Alaska, Canadian provinces and territories. (1) Number of species previously recorded based on Yu et al. (2005) and Fernández-Triana et al. (2009b). (2) Number of species recorded in the present paper. (3) Increase in the species number (%).

	AB	AK	BC	MB	NB	NL	NS	NT	NU	ON	PE	QC	SK	YT	ALL
(1)	25	13	45	10	26	9	22	6	1	80	3	47	8	1	151
(2)	38	16	73	57	46	20	38	10	4	136	8	97	16	3	225
(3)	52	23	62	470	77	112	73	67	300	70	167	106	100	200	50

Although the increase in species numbers is significant, many gaps still remain in our understanding of the group in Canada/Alaska. For example, the list of species for the northern areas (AK, NT, NU and YT), the Atlantic provinces and the Prairies are far from complete; and studies currently underway should increase significantly the numbers provided in this paper. Similarly, the examination of specimens housed in western Canadian collections will be necessary if progress is to be made in BC, AB and SK.

**Figure 1.**
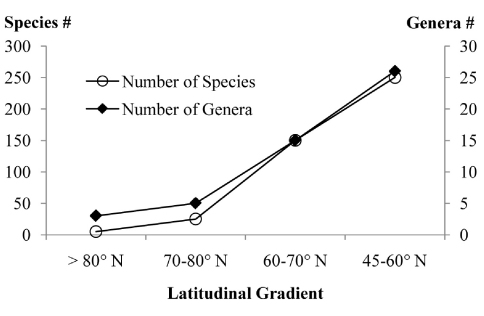
Latitudinal gradients in the species and genera richness of Microgastrinae from Canada and Alaska. Figures based on this paper and work in progress.

Based on this paper and work in progress, the latitudinal gradient of species and genera richness within the studied area show a marked increase towards south (Fig. 1), as would be expected. North of 80° N (northern tip of Ellesmere Island) there are only 4–5 species in 3 genera of Microgastrinae. Between 70–80° N (most of the Canadian Arctic Archipelago with a few areas from the mainland, comprising almost exclusively tundra) there are 20–25 species from about 5 genera. Within the latitudinal range of 60–70° N (most of Alaska and the three Canadian territories, comprising mostly boreal forest with some tundra) there are at least 150 species and 15 genera (e.g. Fernández-Triana et al. 2009a). The southernmost range considered here (45–60° N, comprising the rest of Canada with many ecoregions represented) has over 250 species in 26 genera, but these figures are less conclusive because many more species await to be recorded –and thus should be seen as an underestimate.

**Figure 2.**
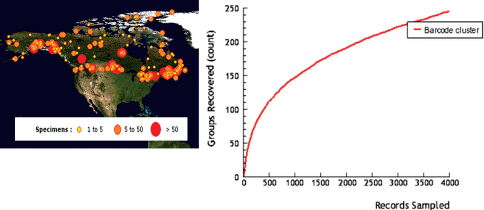
Localities of specimens (**A**), and cumulative number of species (**B**), of Microgastrinae from Canada and Alaska sampled for DNA barcoding. Based on data from BOLD (see Methods for more details).

The biogeographical affinities of the fauna can be analyzed from the distributional data detailed in the checklist below. If only the described species (197 in total) are accounted for, 67% are widely distributed in the Nearctic, especially in Eastern North America (the latter could be an artefact due to the more intensive studies and efforts done in that area); 15% are Holarctic species, many of them intentionally introduced for Biological Control programs; 10% are strict Canada/Alaska endemics (which is equivalent to say that they are restricted to the northern part of the Nearctic region); 4% of the species are also found in the Neotropics; and 4% are cosmopolitan.

The most diverse genera are Cotesia, Apanteles, Microplitis, Pholetesor and Dolichogenidea, while Microgaster, Glyptapanteles and Diolcogaster also have significant, though smaller, number of species. Of these, only Pholetesor has been recently revised (Whitfield 2006) and its figure should be close to the actual number of species expected in the region (but see the checklist below). For the other genera (and especially for Cotesia, Microplitis, Apanteles, Dolichogenidea and Glyptapanteles), the figures provided here represent just a fraction of the actual diversity; with many more undescribed species among the CNC holdings as well as some that have been recently listed (e.g. Fernández-Triana et al. 2009b). These records are not considered in this paper because comprehensive taxonomic reviews are needed to unravel the true magnitude of Microgastrinae in the region, a daunting task that will require years of work.

It is difficult to provide accurate estimates of the actual diversity of the subfamily when so many species await study. However; the analysis of the available DNA barcoding data, the revision of the collections made so far, and the information of well studied areas (see below) suggest that the actual diversity of Microgastrinae in Alaska/Canada will be at least twice the number recorded in the present paper.

There are currently over 3,500 specimens of microgastrine wasps in BOLD with CO1 sequences, collected from localities all over Canada and Alaska (Fig. 2A). In spite of the relatively small proportion of specimens barcoded (compared to the more than 30,000 specimens from Canada and Alaska available in the collections studied) they represent over 240 species (Fig. 2B), an astonishing figure that surpasses the total of species listed in the present paper. DNA barcoding has proven to be a reliable tool to separate species of Microgastrinae (e.g. Smith et al. 2008), especially when supplemented by critical natural history data, and has tremendous potential to help reveal cryptic species for such a diverse subfamily.

The proportion of Lepidoptera to Microgastrinae from three well known areas within the region was also calculated (Table 2) and then the average was extrapolated to estimate the total of Microgastrinae for Alaska/Canada. Choosing Lepidoptera makes sense because they are a much better known group and, most importantly, they are the hosts of Microgastrinae, which parasitizes almost all of the lepidopteran families (Whitfield 1997). The proportion of host/parasitoid species was between 7 and 17, with an average of 12. Interestingly, the same proportion (10–12) is found in other well studied areas around the world such as temperate British Isles and tropical Area de Conservacion de Guanacaste, Costa Rica –data calculated from Fauna Europaea (van Achterberg 2004) and Janzen et al. (2009) respectively. If a proportion of 12 Lepidoptera to each Microgastrinae is extrapolated to the all Canada/Alaska fauna -with over 6700 estimated species of Lepidoptera (Biological Survey of Canada 2010), the results show an estimated diversity of about 550 species of Microgastrinae for the region.

**Table 2.** Number of Lepidoptera and Microgastrinae species in selected areas of Canada. The figures are rounded to the nearest tenth for Microgastrinae species and to the nearest integer for the L/M ratio. For Lepidoptera the data are taken from Danks (1981) for the Arctic Archipelago; Lafontaine and Wood (1997) for the Yukon; and Lafontaine (1997) for Ottawa. Microgastrinae figures are based on the present paper and unpublished data of the author.

Area	Latitude (Area in km2)	# Lepidoptera (L)	# Microgastrinae (M)	L/M Proportion
Canadian Arctic Archipelago	>70° N (1,400,000 km2)	136	20	7
Yukon Territory	60–70° N (475,000 km2)	~2,000	120–150	13–17
Ottawa and surroundings	45° N (~8,000 km2)	2,068	150–200	10–14

Regardless of the approach used, even the most conservative scenarios show an unexpected and unprecedented level of species diversity in a region of the planet supposed to have a rather low diversity. The results reported here, as well as previous papers from other areas (e.g. Smith et al. 2008) suggest that indeed the Microgastrinae might be much more diverse than anticipated.

## Description of new species

### Apanteles huberi

Fernández-Triana sp. n.

urn:lsid:zoobank.org:act:D245A5D6-6061-46B0-A0FA-9271F0DB82DE

**Figures 3–5.**
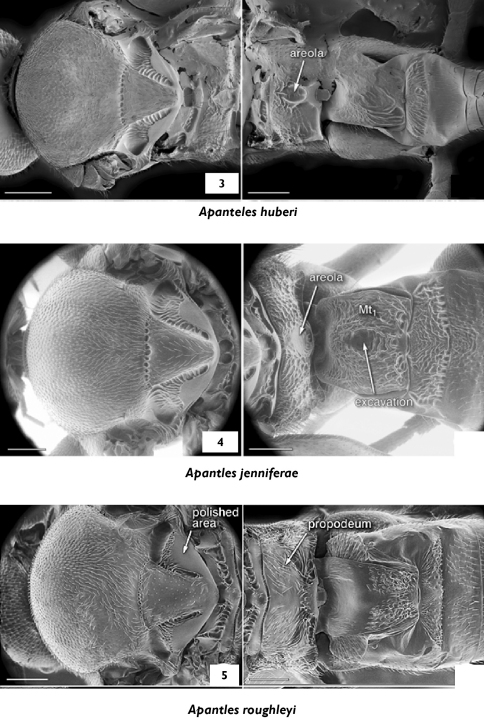
**3** Apanteles huberi, mesosoma, propodeum and medio tergites 1–3, dorsal **4** Apanteles jenniferae, propodeum and medio tergites 1–3, dorsal **5** Apanteles roughleyi, propodeum and medio tergites 1–3, dorsal.

Figs 3 6 Supplementary Appendix 1

Apanteles  sp. 2. Fernández-Triana and Huber, 2010: 316. [Examined].

#### Type locality.

Canada, British Columbia, Kispiox, 55°21'0"N, 127°40'58.8"W.

#### Type material.

##### Holotype.

**Female** (CNC), with first label: Choristoneura biennis, Kispiox, BC, T. G. Gray; second label with date as follows: 6.vii.1983; third label with Specimen ID: MIC 000108. CNC TYPE 23935.

##### Paratypes

(CNC):8 ♀ and 2 ♂ same data as holotype for the first two labels; 4 of those specimens with a third label with Specimen IDs: MIC 000106 and MIC 000107 (2 ♀), MIC 000109 (1 ♂), and CNCI JDR-specm 2009–470 (1 ♀).

#### Diagnosis.

This species will run to Apanteles fumiferanae in both the keys of Muesebeck (1922) and Mason (1974); and will run to Apanteles sp. 2 in the key of Fernández-Triana and Huber (2010). It is related to and morphologically very similar to Apanteles fumiferanae. It differs in the propodeal areola shape (elongated coffin-shaped or ovoid, and weakly defined anteriorly in Apanteles huberi; less elongated and well defined diamond-shaped in Apanteles fumiferanae), length of flagellomeres 2 and 14 (longer in Apanteles huberi) and medio tergite 1 (in Apanteles huberi almost parallel-sided, with greatest width 1.1× the shortest width; while in Apanteles fumiferanae the medio tergite is widening apically, with the greatest width 1.2–1.5× the shortest width). The two species also have different host species and differ in 1–4 base pairs within the barcoding region (more details below under the sections **Molecular data**, **Distribution and biology** and **Comments**).

#### Description.

##### Female

Antenna length 2–2.2 mm (missing in holotype), body length 2.7 mm (2.3–2.8 mm), forewing 2.8 mm (2.6–3.0 mm). Head with glossa truncate and short. Face with shallow, sparse punctures; and sparse, uniformly distributed setae. Face width at antennal base/face width at clypeus edge: 1.1×; intertentorial pit distance/face width at clypeus edge: 0.6×; compound eye height/head height: 0.8×; head height/width: 0.8×; face width at antennal base/head maximum width: 0.7×; malar space/basal width of mandible 1.1×. Clypeus transversely narrow, its width/height: 3.7×. Length/width of flagellomeres: 1^st^ (3.5×), 2^nd^ (4.0×), 8^th^ (2.9×), 14^th^ (1.2×), 15^th^ (1.1×). Length of flagellomere 2/flagellomere 14: 3.0×. Ocello-ocular distance/posterior ocelli diameter: 2.3×; distance betwen posterior ocelli/ocelli diameter: 2.3×.

Mesosoma. Pronotum laterally with dorsal and ventral grooves well defined. Mesoscutum with sparse and shallow punctures (distance between punctures about 1.0× its diameter), punctures sparser centrally. Mesoscutum 1.4× wider than long. Mesoscutum and scutellum uniformly covered by dense, silvered-coloured pilosity. Scutellum almost smooth, with very sparse and shallow punctures. Scutellum length/width at base 1.1×. Scutellar suture thin and shallow, with 12–14 costulae. Posterior band of scutellum polished. Scutellar lateral face with polished area semicircular and about 1/2 the face height. Mesopleuron setose and with punctures on the anterior margin and upper corner, rest smooth and glabrous; centrally with small depressed area with shallow transverse striae. Thin, crenulate sulcus separating meso and metapleura. Metapleuron mostly smooth and polished, with setae and punctures only dorsally and ventrally along margins; metapleuron with a short, crenulate, longitudinal sulcus running from lower margin near metacoxa through spiracle. Metapleural carina with a short lamella. Propodeum with an ovoid or coffin-shaped areola, with anterior carinae less defined; propodeum sparsely punctured in the anterior half, with transverse striation in the apical half.

Metasoma. Mediotergite 1 almost parallel sided, just slightly widening posteriorly; basal width/apical width 1.1×; length/apical width 1.4×; mediotergite 1 with smooth, basal depression; apical 2/3 sculptured with longitudinal striae, except for a median, sub-apical depressed area which is mostly smooth and a polished knob centrally in the apical margin. Mediotergite 2 transverse, trapezoidal in shape; basal width/apical width 0.7×; length/apical width 0.3×; with longitudinal striae covering most of the surface. Mediotergite 3 twice the length of mediotergite 2. Mediotergite 3 and following unsculptured, polished and uniformly covered by sparse setae. Hypopygium striate, with acute tip slightly protruding beyond apical tergites. Ovipositor sheaths fully setose, 0.9–1.0× as long as metatibia length.

Legs. Metatibial inner spur 1.3× (1.2–1.5×) the length of outer spur, and 0.6× (0.5–0.6×) the length of metatarsomere 1. Metafemur 3.0× (3.0–3.1×) as long as wide.

Wings. Forewing vein R1a 1.1× as long as stigma length; length of R1a about 2.0× as long as the distance between its end and the end of 3RSb. Vein r 0.8× the maximum width of stigma. Join of veins r and 2RS angulated, sometimes with small knob at their junction; vein 2M 1.0–1.1× as long as vein (RS+M)b. Edge of vannal lobe of hindwing medially straight to slightly convex and with setae of uniform length which are shorter than those at base and apex of lobe.

Colour: Maxillary and labial palps, and two first pairs of legs (except for coxae), yellow; head, meso and metasoma, and all coxae dark-brown or black; apex of metatibia and part (sometimes most) of the metafemur and metatarsus orange-red or light brown. Most of veins light brown, stigma borders light brown, centrally pale.

##### Male

As females, except for slightly smaller size (2.3–2.4 mm), legs with brighter yellow coloration, and width of mediotergite 1 slightly less than in females.

**Figures 6–8.**
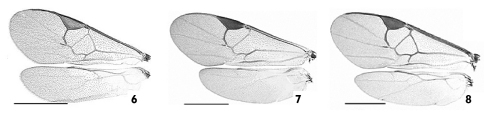
Wings. **6** Apanteles huberi **7** Apanteles jenniferae **8** Apanteles roughleyi. Scale line= 1.0 mm.

#### Molecular data.

Partial barcodes (144 bp) from the holotype and three paratypes of Apanteles huberi were obtained and compared with two paratypes of Apanteles fumiferanae with a similar sequence length (Fig. 21). In spite of the relatively short sequences available for analysis (about one fifth of the barcoding region) the two species consistently differed between 1–4 base pairs (0.8–2.8%).

#### Distribution and biology.

Only known from the type locality in BC. All studied specimens were reared from Choristoneura biennis —it is the only braconid species reliably reared from that lepidopteran (Fernández-Triana and Huber 2010).

#### Comments.

The related species Apanteles fumiferanae has a relatively wide range of hosts (Mason 1974; Fernández-Triana and Huber 2010), but has never been recorded parasitizing Choristoneura biennis. The different host species and slight but consistent morphological and barcoding differences provide sufficient evidence to consider Apanteles huberi as a separate and distinct species.

#### Etymology.

I dedicate this species to John Huber (CNC) as an appreciation for the many things I have learned from him during the last four years (his knowledge of Hymenoptera and kindness are both extraordinary); and also for all the shared chocolate!

### Apanteles jenniferae

Fernández-Triana sp. n.

urn:lsid:zoobank.org:act:B57489C7-8EC6-4513-A918-0844DF48BF45

Figs 4 7

Apanteles  sp. 1. Fernández-Triana and Huber, 2010: 316. [Examined].

#### Type locality.

Canada, New Brunswick, Canterbury, 45°53'20.5"N, 67°27'49.6"W.

#### Type material.

##### Holotype.

**Female** (CNC), with first label as follows: C-26, Ex Choristoneura rosaceana Harr. on Red Maple; second label: Canterbury, York Co., N.B., 6.vii.1973. CNC TYPE 23936.

##### Paratypes

(CNC): 3 ♀, 2 ♂ from Canterbury, NB; Galetta, Delta, and North Bay, ON; Old Chelsea and Tenoga, QC; ex: Choristoneura rosaceana (CNC).

#### Diagnosis.

This species is related to Apanteles fumiferanae but it is differentiated by its slightly larger size; yellow tegula; less defined areola (mostly marked by a depression and with only apical carinae; contrasting with a complete areola, well defined by carinae in Apanteles fumiferanae); medio tergite 2 (less transverse in Apanteles jenniferae, thinner in Apanteles fumiferanae); and meditergite 3 (Apanteles jenniferae with some sculpture centrally in anterior margin basally, completely smooth in Apanteles fumiferanae).

#### Description.

##### Female

Antenna length 2.2–2.3 mm, body length 3.1 mm (3.0–3.4 mm), forewing 3.2 mm (3.2–3.6 mm). Head with glossa truncate and short. Face with shallow punctures (separation between punctures about the same than punctures diameter); and sparse, uniformly distributed setae. Face width at antennal base/face width at clypeus edge: 1.6×; intertentorial pit distance/face width at clypeus edge: 0.6×; compound eye height/head height: 0.7×; head height/width: 0.8×; face width at antennal base/head maximum width: 0.5×; malar space/basal width of mandible 1.3×. Clypeus transversely narrow, its width/height: 3.5×. Length/width of flagellomeres: 1^st^ (3.9×), 2^nd^ (3.8×), 8^th^ (3.0×), 14^th^ (1.4×), 15^th^ (1.2×). Length of flagellomere 2/flagellomere 14: 2.6×. Ocelo-ocular distance/posterior ocelli diameter: 2.0×; distance betwen posterior ocelli/ocelli diameter: 2.0×.

Mesosoma. Pronotum laterally with dorsal and ventral grooves well defined. Mesoscutum with relatively close punctures (distance between punctures about 0.5× its diameter). Mesoscutum 1.4× wider than long. Mesoscutum and scutellum uniformly covered by dense, silvered-coloured pilosity. Scutellum almost smooth, with very sparse and shallow punctures mostly on the margins. Scutellum length/width at base 1.0×. Scutellar suture well impressed, with 12 costulae, the central ones more spaced and deeply impressed than the lateral ones. Posterior band of scutellum polished. Scutellar lateral face with polished area semicircular slightly less than half the face height. Mesopleuron setose and with punctures on the anterior half; the posterior half glabrous and smooth except for a thin sulcus running from the posterior margin (at about half the length of that margin) towards the lower margin of mesopleuron (ending just before the punctures and setae of the anterior half). Thin, crenulated sulcus separating meso and metapleura. Metapleuron mostly smooth and polished, with setae and punctures only dorsally and ventrally along margins; metapleuron with a very short, crenulate, longitudinal sulcus running from lower margin near metacoxa through spiracle. Metapleural carina with short lamella. Propodeum with areola defined mostly by a central impression than carinae -though the posterior carinae are visible; propodeum coarsely punctured in the anterior half, with transverse striation in the apical half, the only smooth area is centrally inside the areola.

Metasoma. Mediotergite 1 barrel-shaped, wider medially than anteriorly or posteriorly; basal width/apical width 0.9× (0.8–0.9×); length/apical width 1.1×; mediotergite 1 with smooth, basal depression; apical 2/3 coarsely sculptured and with longitudinal striae, except for a median, sub-apical depressed area which is mostly smooth and a polished knob centrally in the apical margin. Mediotergite 2 transverse, trapezoidal to almost rectangular in shape; basal width/apical width 0.7×; length/apical width 0.3×; coarsely sculptured with longitudinal and transverse striae covering most of the surface, the posterior margin bordered by distinct, crenulated punctures. Mediotergite 3 about 1.5× the length of mediotergite 2 and with some sculpture centrally in the anterior margin. Mediotergite 4 and following unsculptured, polished and uniformly covered by sparse setae. Hypopygium striate, with acute tip slightly protruding beyond apical tergites. Ovipositor sheaths fully setose, 1.0× (0.9–1.1×) as long as metatibia length.

Legs. Metatibial inner spur 1.4× (1.4–1.6×) the length of outer spur, and 0.6× (0.5–0.6×) the length of metatarsomere 1. Metafemur 3.0× (3.0–3.2×) as long as wide.

Wings. Forewing vein R1a 1.0–1.1× as long as stigma length; length of R1a 6–7.0× as long as the distance between its end and the end of 3RSb. Vein r 1.0× (1.0–1.1×) the maximum width of stigma. Join of veins r and 2RS angulated and with a small knob at their junction; vein 2M 0.8× (0.7–0.9×) as long as vein (RS+M)b. Edge of vannal lobe of hindwing medially straight to convex and glabrous.

Colour: Maxillary and labial palps, tegula, two first pairs of legs (except for coxae), and basal half of metafemur yellow; head, meso and metasoma dark-brown or black; wing base and all coxae brown; metafemur, apical half of metatibia and metatarsus yellowish- brown to orange-brown. Most of veins very light brown, almost hyaline; stigma light brown.

##### Male

As female except for longer flagellomere, antenna longer than body length, darker hind legs (with metafemur dark brown), and less transverse medio tergite 2 (which is almost quadrate and with striation arranged in a concentric way).

**Figures 9–12.**
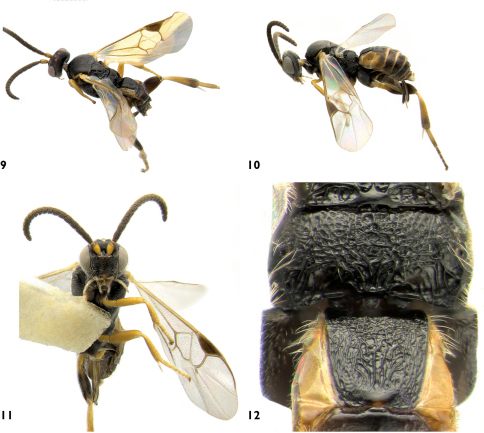
Apanteles samarshalli. **9** Dorsolateral **10** Lateral **11** Ventral **12** Propodeum and medio tergites 1–2.

#### Distribution and biology.

The species is widely distributed in eastern Canada, where it has been reared from Choristoneura rosaceana.

#### Comments.

This species and the previous one (Apanteles huberi) illustrate well the need for a review of what Fernández-Triana and Huber (2010) called “the Apanteles fumiferanae species-complex”. It is becoming obvious that many species are hidden under that name, and a comprehensive approach combining detailed morphology, biology (especially verified host records) and molecular data will be required to unravel the rest of the species within that complex.

#### Etymology.

I dedicate this species to Jennifer Read (CNC) to thank her for the many hours she spent taking photos for several projects we worked upon together; and as recognition for her superb photographic and editing skills.

### Apanteles masmithi

Fernández-Triana sp. n.

urn:lsid:zoobank.org:act:28705735-DE76-4BDD-AC43-65DF217462F2

**Figures 13–16.**
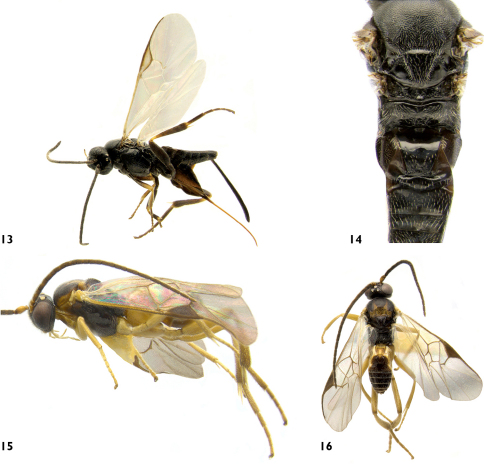
**13** Apanteles masmithi, lateral **14** Apanteles masmithi, meso and metasoma, dorsal **15** Distatrix carolinae, lateral **16** Distatrix carolinae, dorsal.

Figs 13, 14 Supplementary Appendix 1 

#### Type locality.

Canada, Ontario, London, 42°59'1.32"N, 81°14'58.92"W.

#### Type material.

##### Holotype.

Female (CNC), with first label as follows: London, ON, 23.viii.1953, W.W. Judd, on Typha heads; second label (yellow) with a code: 54-B-4; third label with a provisional identification by Mason (1955); fourth label with Specimen ID: MIC 000048. CNC TYPE 23937.

##### Paratypes

(CNC): 3 #F from London, ON, 23.viii.1953, W.W. Judd, on Typha heads, one of those specimens with a third label with Specimen IDs: MIC 000049; 5 #F, 6 #M from Digby, NS, 28.viii.1959, P.H.H. Gray, ex Limnaecia phragmitella, four of those specimens with a third label with Specimen IDs: MIC 000050, MIC 000051, MIC 000052 (3 #F), and MIC 000054 (1 #M); 5 #F, 1 #M from Lunenburg, NS, vi-vii.1969, B. Wright, ex Gelechidae larvae on cat-tail heads, two of those specimens with a third label with Specimen IDs: MIC 000053 (1 #F), and MIC 000057 (1 #M); 2 #F, 4 #M, Brighton, NS, P.H.H. Gray, ex. Limnaecia phragmitella and Dycimotomia julianalis, on Typha heads, two of those specimens with a third label with Specimen IDs: MIC 000055 and MIC 000056 (2 #M); 1 ♂, Leeds-Granville Co., forest, ix-x.2008, S. B. Peck. Specimen ID: CAM 0456.

#### Diagnosis.

This species looks similar to Apanteles cockerelli Muesebeck, 1921; and it will run to that species in the available keys (e.g. Muesebeck 1921). It differs in the stigma colour in the fore wing (pale with only brown borders in Apanteles masmithi, completely brown in Apanteles cockerelli); the shape of vannal lobe in the hind wing (straight and with short setae medially in Apanteles masmithi, concave and glabrous in Apanteles cockerelli), and the relative length of the metatibial spurs (about the same length in Apanteles masmithi, the inner spur longer than the outer one in Apanteles cockerelli). The two species also have different geographic distribution, different host species, and differ in 14 base pairs within the barcoding region (more details below under the sections **Molecular data**, **Distribution and biology** and **Comments**).

#### Description.

##### Female

Antenna length 2.6 mm (2.1–2.5 mm), body length 3.7 mm (2.8–3.6 mm), forewing 3.5 mm (2.6–3.5 mm). Head with glossa bilobated and rather long. Face smooth, with very shallow punctures (separation between punctures larger than punctures diameter) and very sparse setae. Face width at antennal base/face width at clypeus edge: 1.1×; intertentorial pit distance/face width at clypeus edge: 0.6×; compound eye height/head height: 0.7× (0.6–0.7×); head height/width: 0.9×; face width at antennal base/head maximum width: 0.6×; malar space/basal width of mandible 1.7× (1.3–1.7×). Clypeus not much transverse, its width/height: 2.6×. Length/width of flagellomeres: 1^st^ (3.1×), 2^nd^ (3.1×), 8^th^ (2.3×), 14^th^ (1.3×), 15^th^ (1.0×). Length of flagellomere 2/flagellomere 14: 2.2×. Ocelo-ocular distance/posterior ocelli diameter: 2.3× (1.9–2.3×); distance betwen posterior ocelli/ocelli diameter: 2.7× (2.1–2.7×).

Mesosoma. Pronotum very smooth and polished, laterally with dorsal and ventral grooves thin but deep and well defined. Mesoscutum mostly smooth, with shallow punctures (distance between punctures about its diameter), punctures a little closer and deeper in the posterior margin. Mesoscutum 1.2× (1.1–1.2×) wider than long. Mesoscutum and scutellum covered by sparse, silvered-coloured pilosity. Scutellum almost smooth, with very sparse (distance between punctures twice its diameter) and shallow punctures concentrated mostly on the margins. Scutellum length/width at base 1.1×. Scutellar suture thin and shallow, with 16 (15–17) costulae. Posterior band of scutellum polished. Scutellar lateral face with the polished area triangular and about 4/5 the face height. Mesopleuron smooth and glabrous on most of its surface, with sparse setae and punctures (distance between punctures twice or more its diameter) only on the anterior and dorsal margins. Thin and shallow sulcus, with a few costulae, separating meso and metapleura. Metapleuron mostly smooth and polished, with setae and sparse punctures only dorsally and posteriorly along margins; metapleuron with a thin, longitudinal sulcus running from lower margin through spiracle. Metapleural carina with short lamella. Propodeum mostly smooth, with sparsely punctures in the anterior half and a few transverse striae in the apical half; propodeal areola absent but there is a short, postero-median longitudinal band of rugosity (consisting of several very short carinae radiating from nucha).

Metasoma. Mediotergite 1 arched and strongly narrowing toward apex, with a wide and deep basal depression; basal width/apical width 2.2×; length/apical width 2.3 (2.0–2.3×); mediotergite 1 mostly smooth, polished and glabrous, with a few setae and elongated, longitudinal punctures postero-laterally. Mediotergite 2 smooth and polished, transverse, and wider centrally; basal width/apical width 1.0× (0.9–1.0×); length/apical width 0.5× (0.3–0.5×). Mediotergite 3 2.0× (2.0–2.5×) the length of mediotergite 2. Mediotergite 3 and following unsculptured, polished and uniformly covered by setae. Hypopygium striate, with acute tip protruding beyond apical tergites. Ovipositor sheaths fully setose, 1.9× (1.8–1.9×) as long as metatibia length.

Legs. Metatibial inner spur about the same length of outer spur, and 0.4× (0.4–0.5×) the length of metatarsomere 1. Metafemur 2.8× as long as wide.

Wings. Forewing vein R1a 1.0× as long as stigma length; length of R1a 5.0× as long as the distance between its end and the end of 3RSb. Vein r 0.8× the maximum width of stigma. Join of veins r and 2RS angulated and with a small know marking the angulation (sometimes only slightly angulated and then know very small to absent); vein 2M 0.6× as long as vein (RS+M)b. Edge of vannal lobe of hindwing medially straight and with short setae that are slightly sparser than the rest of the lobe.

Colour: Mostly black to dark brown, except for: maxillary and labial palps (light brown to brown), wing base (light brown), profemur and part of most of all tibia and tarsi (light brown to yellow), meso and metatibial spurs (light yellow to witish). Wings hyaline, with most of veins transparent, except for C+Sc+R, R1, and occasionally r and 2RS which can be partially pigmented; stigma hyaline except for brownish borders.

##### Male

Similar to females but slightly smaller in size and with longer antennal segments (especially the apical ones). The maxillary and labial palpi tend to be yellow, and the legs tend to be darker (mostly black with less yellow areas). The mediotergite 1 is fully smooth and polished, and narrows stronger toward apex (being thinner compared to that of females). The wing veins are paler, of milky coloration, including the stigma (which brown borders are very thin, almost disappearing in some specimens).

#### Variation.

There is some variation in size among the different localities (it is shown in the description) and also the maxillary and labial palpi range from dark brown to yellow.

#### Molecular data.

Barcodesof 6 specimens of Apanteles masmithi and 3 of the related species Apanteles cockerelli were compared. Because all specimens but one were collected between 1951 and 1969 it was only possible to obtain mini-barcodes (144 bp). The only recent specimen (a paratype of Apanteles masmithi, collected in 2008) rendered a full barcode (657 bp) which fully matched the other specimens with mini-barcodes. The molecular results confirmed that they are indeed different species, with at least 14 (9.7%) of base pairs divergence (Fig. 22). Interestingly, specimens of Apanteles cockerelli within the US (from CA, MO and TX) seem to comprise more than one species -but that is beyond the geographical scope of the present work, thus they will be dealt with in a different paper.

#### Distribution and biology.

The species is widely distributed in Eastern Canada, where it has been recorded parasitizing Limnaecia phragmitella (Gelechidae) on Typha spp. heads (cattail grass). Some paratypes from Nova Scotia had written on their labels that the host could also be Dycimotomia julianalis (Pyralidae), also on cattail; however, this record needs to be confirmed. This is the first Microgastrinae (and Braconidae) species recorded as parasitoid of Limnaecia phragmitella.

#### Comments.

The specimens of Apanteles masmithi were identified by W. Mason as a different but related species to Apanteles cockerelli. The latter species has been recorded from US in the following ten states: CA, IA, ID, MI, MO, NE, NM, OR, SD, TX (Yu et al. 2005). The different host species (Isophrictis sp. (Gelechidae) for Apanteles cockerelli), 14 (9.7 %) of base pairs divergence within the barcoding region, and slight but consistent morphological differences, provide sufficient evidence to consider Apanteles masmithi as a distinct species.

#### Etymology.

I dedicate this species, which DNA barcoding helped to recognize, to M. Alex Smith (University of Guelph) as an appreciation for the many parasitoid wasps he has helped to barcode, study and publish about; and also for sharing with me his superb knowledge on molecular approaches.

### Apanteles roughleyi

Fernández-Triana sp. n.

urn:lsid:zoobank.org:act:E392BA33-CAC1-40F2-ADE5-440D4B017969

Figs 5 8

Apanteles  sp. near *stagmatophorae*. Fernández-Triana and Huber, 2010: 316. [Examined].

#### Type locality.

Canada, British Columbia, Mill Bay, 48°39'2"N, 123°33'33"W.

#### Type material.

##### Holotype.

**Female** (CNC), with first label with Specimen ID: CNCI JDR-specm 2009–463; second label with Forest Insect Survey number: 65.21.01A, and date: 22.iii.1965; third label as follows: Apanteles grandis [probably Abies grandis], Mill Bay, B.C.; fourth label: Ex? Choristoneura fumiferanae; fifth label with a provisional identification by Mason 1978. CNC TYPE 23938.

#### Diagnosis.

This species looks similar to Apanteles stagmatophorae Gahan, 1919, and it will run to that species in the available keys (e.g. Muesebeck, 1921), but they differ in several characteristics. In Apanteles roughleyi the vannal lobe of hindwing is medially straight and glabrous (slightly convex to slightly straight but with uniform setae in Apanteles stagmatophorae), the ovipositor sheaths are longer (1.7× compared to 1.2×), the metafemur is thinner (3.5× as long as wide compared to 3.2×), and the propodeum is more sculptured (in Apanteles stagmatophorae the propodeum is mostly smooth, with very shallow and small punctures). The two species have a very separate distribution (BC in western Nearctic for Apanteles roughleyi; Maryland, in eastern Nearctic for Apanteles stagmatophorae). The known host are also from different families: Choristoneura sp., Tortricidae, for Apanteles roughleyi; Periploca gleditschiaeella (Chambers, 1876), Cosmopterigidae, for Apanteles stagmatophorae (more details below in the section **Distribution and biology**).

#### Description.

##### Female

Antenna broken, body length 3.3 mm, forewing 3.5 mm. Head with glossa truncate and short. Face with shallow punctures (separation between punctures about the same than punctures diameter) and uniformly distributed setae. Face width at antennal base/face width at clypeus edge: 1.0×; intertentorial pit distance/face width at clypeus edge: 0.5×; compound eye height/head height: 0.8×; head height/width: 0.8×; face width at antennal base/head maximum width: 0.7×; malar space/basal width of mandible 1.2×. Clypeus transversely narrow, its width/height: 3.0×. Length/width of flagellomeres: 1^st^ (2.6×), 2^nd^ (2.2×), 8th (2.3×), flagellomeres 12+ missing. Ocelo-ocular distance/posterior ocelli diameter: 2.0×; distance betwen posterior ocelli/ocelli diameter: 2.0×.

Mesosoma. Pronotum laterally with dorsal and ventral grooves thin, but well defined and deep. Mesoscutum with very shallow and sparse punctures (distance between punctures 1.5–2.0× its diameter). Mesoscutum 1.4× wider than long. Mesoscutum uniformly covered by silvered-coloured pilosity; scutellum almost glabrous, with just a few setae on margins. Scutellum almost smooth, with very sparse, small and shallow punctures mostly on the center. Scutellum length/width at base 1.1×. Scutellar suture very thin and shallow, with about 20 small and not well defined costulae. Posterior band of scutellum polished. Scutellar lateral face with polished area semicircular about 0.6× the face height. Mesopleuron setose and with sparse punctures only on the anterior margin; the rest glabrous, smooth and polished. Thin, crenulated sulcus separating meso and metapleura. Metapleuron mostly smooth and polished, with setae and punctures only dorsally and ventrally along posterior margin; metapleuron with a thin sulcus running from lower margin near metacoxa through spiracle. Metapleural carina with a very short lamella. Propodeum mostly punctured, with a few striae postero-laterally; propodeal areola absent, but there is a central smooth area (contrasting with rest of the propodeum sculpture) and also there is a short, postero-median longitudinal band of rugosity (consisting of several short carinae radiating from nucha).

Metasoma. Mediotergite 1 narrowing towards apex; basal width/apical width 1.6×; length/apical width 1.9×; mediotergite 1 with smooth, basal depression; apical half coarsely punctured, except for a polished knob centrally in the apical margin. Mediotergite 2 transverse, trapezoidal in shape; basal width/apical width 0.5×; length/apical width 0.2×; sculptured with longitudinal striation and puntures covering most of the surface except the center. Mediotergite 3 1.6× the length of mediotergite 2. Mediotergite 3 and following unsculptured, polished and uniformly covered by setae. Hypopygium striate, with an acute tip protruding well beyond the apical tergites. Ovipositor sheaths fully setose, 1.7× as long as metatibia length.

Legs. Metatibial inner spur 1.1× the length of outer spur, and 0.5× the length of metatarsomere 1. Metafemur 3.5× as long as wide.

Wings. Forewing vein R1a 1.3× as long as stigma length; length of R1a 5.7× as long as the distance between its end and the end of 3RSb. Vein r 1.0× the maximum width of stigma. Join of veins r and 2RS slightly angulated; vein 2M 1.1× as long as vein (RS+M)b. Edge of vannal lobe of hindwing medially straight and glabrous.

Colour: Mostly black to dark brown, except for: maxillary and labial palps (yellow); tegula and wing base (light brown); first two pairs of legs (yellow except for coxae which are partially light brown); hind legs (mostly yellow-brown, with metacoxa brown and dorsal brown marks on metafemur, metatibia and metatarsi). Wings hyaline, with most of veins brown, including stigma.

##### Male

Unknown.

#### Distribution and biology.

The host information (Choristoneura fumiferana) was recorded originally in 1965, i.e., before Freeman (1967) split the genus Choristoneura and changed the species boundaries. The actual host is either Choristoneura occidentalis or Choristoneura pinus, but there is no way to determine which.

#### Comments.

The specimen bears a label by W. Mason, dated 1978, stating that it may actually be a new species related to Apanteles stagmatophorae. Comparison with two paratypes of the later species (housed in the CNC) confirms that the two species are distinct. In spite of the fact there is only one known specimen, the species is described to provide a name because of its potential economic importance (Fernández-Triana and Huber 2010).

#### Etymology.

I dedicate this species to the late Rob Rougley (University of Manitoba) who passed away when this paper was starting. We all miss you dear friend and colleague, but I am sure you should be chasing heavenly Ditiscidae beetles right now!

### Apanteles samarshalli

Fernández-Triana sp. n.

urn:lsid:zoobank.org:act:D62FD0A7-E529-4162-8233-49C471073C64

Figs 9–12 Supplementary Appendix 1 

#### Type locality.

United States, Florida, Monroe County, Key Largo, 25°5'11.4"N, 80°26'50.28"W.

#### Type material.

##### Holotype.

**Female** (CNC), with first label: FLA: Monroe Co., N. Key Largo, secondary hammock forest, iii-iv.1985; second label with Specimen ID: CNCH1234. CNC TYPE 23939.

##### Paratypes

(CNC): 2 ♀ from N. Key Largo, Monroe Co., FL, secondary hammock forest, iii-iv.1985; 2 ♀ from Fat Deer Key, Monroe Co., FL, iii-iv.1985; 1 ♀ from Everglades National Park, Royal Palm Hammock, Monroe Co., FL, hammock forest, iii-iv.1985, S & J. Peck; 2 ♀ from Archbold Biological Station, Highlands Co., FL, 26.iv.1967, B. V. Peterson; 1 ♀ from Rondeau Prov. Pk, ON, Mal. Trap, 19.viii-11.ix.1973.

#### Diagnosis.

Thus far this is the only Nearctic species of Apanteles with a significantly short antenna (half the body length); vein 2M very short, almost obliterating with vein 2RS; and antenna with yellow scape/pedicel and brown flagellomere. The combination of those characters makes Apanteles samarshalli one of the most distinctive and recognizable species within the genus.

#### Description.

##### Female

Antenna length 1.3 mm (1.3–1.5 mm), body length 2.6 mm (2.5–3.0 mm), forewing 2.3 mm (2.3–2.5 mm). Head with glossa truncate and short. Face with shallow punctures (separation between punctures about the same as its diameter). Face width at antennal base/face width at clypeus edge: 1.0×; intertentorial pit distance/face width at clypeus edge: 0.5×; compound eye height/head height: 0.7×; head height/width: 0.8×; face width at antennal base/head maximum width: 0.6×; malar space/basal width of mandible 1.0×. Clypeus transverse, its width/height: 3.0×. Length/width of flagellomeres: 1^st^ (1.6×), 2^nd^ (1.4×), 8^th^ (0.8×), 14^th^ (0.8×), 15^th^ (0.9×). Length of flagellomere 2/flagellomere 14: 1.9×. Ocelo-ocular distance/posterior ocelli diameter: 1.8×; distance betwen posterior ocelli/ocelli diameter: 1.9×.

Mesosoma. Pronotum laterally with dorsal and ventral grooves well defined. Mesoscutum with coarse, close punctures (distance between punctures less than half its diameter). Mesoscutum 1.2× (1.1–1.2×) wider than long. Mesoscutum and scutellum covered by uniform, large, silvered-coloured pilosity. Scutellum almost smooth, with very shallow and sparse punctures in the margins. Scutellum length/width at base 0.8×. Scutellar suture width 1/6 scutellum length, with 12–14 costulae. Posterior band of scutellum polished. Scutellar lateral face with the polished area triangular and about 4/5 the face height. Mesopleuron with close punctures and setae on the anterior half, smooth and glabrous on the posterior half. Thin and shallow sulcus, with fine costulae, separating meso and metapleura. Metapleuron mostly punctured and with setae, smooth, polished and glabrous only around the spiracle; metapleuron with a longitudinal sulcus running from ventral to dorsal margin of metapleuron through spiracle. Metapleural carina lamellate. Propodeum sculpture reticulate, postero-laterally with longitudinal striation; propodeal areola absent but there is a short, postero-median longitudinal band of rugosity (consisting of several short carinae radiating from nucha).

Metasoma. Mediotergite 1 evenly and slightly narrowing toward apex, with a wide and deep basal depression; basal width/apical width 1.4×; length/apical width 1.5; mediotergite 1 mostly sculptured (except for smooth basal depression and central knob on the posterior margin), with longitudinal striation on its apical 2/3. Mediotergite 2 smooth and polished, transverse, and wider centrally; basal width/apical width 0.8×; length/apical width 0.3×. Mediotergite 3 2.0–2.5× the length of mediotergite 2. Mediotergite 3 and following unsculptured, polished and covered by sparse setae on the posterior margins. Hypopygium striate, with acute tip slightly protruding beyond apical tergites. Ovipositor sheaths fully setose, short, 0.6× as long as metatibia length.

Legs. Metatibial inner spur 1.3× the length of outer spur, and 0.5× the length of metatarsomere 1. Metafemur 2.7× as long as wide.

Wings. Forewing vein R1a 1.3× (1.2–1.5×) as long as stigma length; length of R1a 4.0× (4.0–5.0×) as long as the distance between its end and the end of 3RSb. Vein r about the same length than maximum width of stigma. Join of veins r and 2RS evenly curved, not angulated; vein 2M very short, almost obliterating with 2RS, length of 2M 0.3× as long as vein (RS+M)b. Edge of vannal lobe of hindwing medially strongly concave and glabrous.

Colour: Body black; antenna flagellomere, metacoxa, most of the metafemur and apical ¼ of metatibia brown; mandibles, labrum, maxillary and labial palps, scape, upper corner of pronotum, tegula and laterotergites 1–3, yellow. Wings hyaline, with most of veins brown pigmented; stigma brown with a minute pale spot basally.

##### Male

Unknown.

#### Molecular data.

From all specimens studied, only the holotype rendered a partial sequence (390 bp, approximately 60% of the barcoding region). The specimen matches almost perfectly (99.96%) a Costa Rican species named as Apanteles Rodriguez151 (Smith et al. 2008).

#### Distribution and biology.

The species has been found from the southwestern part of ON (Rondeau Provincial Park, 42°N) to about 25°N in FL (Everglades National Park and Florida Keys). None is know of its host, but most of the specimens have been collected in hammock forests.

#### Comments.

Despite the two widely separate areas of distribution (ON and FL), I have not been able to find any difference between the Canadian and US specimens; thus they are considered as conspecific here. As for the relation with Apanteles Rodriguez151, I have not been able to examine specimens of the latter. If proven con-specific, it would be even more puzzling to explain the distribution of the species. All of those areas share in common the presence of oaks, but the data available is not enough as to draw any solid conclusion at present.

#### Etymology.

I dedicate this species to a great friend and entomologist, Steve A. Marshall (University of Guelph). I hope you have many more collecting and photography trips in the near future!

### Distatrix carolinae

Fernández-Triana sp. n.

urn:lsid:zoobank.org:act:85F029F2-74E5-4EE5-A041-02C9520A0E3B

Figs 15, 16 

#### Type locality.

Canada, Quebec, Gatineau, 45°29'16"N, 75°51'52"W.

#### Type material.

##### Holotype.

**Female** (CNC), with label as follows: Summit King Mtn. Old Chelsea, QUE, 26.vi.77, M. Sandborne. CNC TYPE 23940.

#### Diagnosis.

This species is very similar to Distatrix solanae Whitfield, 1996, the other known Nearctic species. They differ slightly in body coloration (meso and metasoma mostly dark brown in Distatrix carolinae, mostly honey-orange in Distatrix solanae), length/width of flagellomere 2 and 14 (2.9× and 3.3× for Distatrix carolinae and 3.7× and 3.8× for Distatrix solanae respectively) and a longer inner metatibial spur compared to the outer one (1.5× in Distatrix carolinae, 1.2× in Distatrix solanae). The raised medial region of mediotergite 2 is delimited by divergent grooves that fade posteriorly in Distatrix solanae while the grooves are more or less parallel and not fading posteriorly in Distatrix carolinae.

#### Description.

##### Female

Antenna length 3.5 mm; body length 3.2 mm; forewing length 3.7 mm. Head with glossa truncate and short, maxillary and labial palps light yellow. Face with shallow and sparse punctures and uniformly distributed setae. Face width at antennal base/face width at clypeus edge: 1.1×; intertentorial pit distance/face width at clypeus edge: 0.7×; compound eye height/head height: 0.8×; head height/width: 0.8×; face width at antennal base/head maximum width: 0.4×; malar space/basal width of mandible 1.3×. Clypeus transversely narrow, its width/height: 4.6×. Length/width of flagellomeres: 1^st^ (3.0×), 2^nd^ (2.9×), 3^rd^ (3.0×), 8^th^ (3.0×), 14^th^ (3.3×), 15^th^ (3.0×), 16^th^ (3.2×). Ocelo-ocular distance/posterior ocelli diameter: 0.4×; distance betwen posterior ocelli/ocelli diameter: 0.8×.

Mesosoma. Pronotum with ventral groove present, dorsal one almost obliterated. Mesoscutum with shallow, sparse punctures (distance between punctures about the same as its diameter); punctures almost disappearing in the notauli and posterior area of mesoscutum. Notauli not impressed, visible only because of the contrast of different coloration and smoother area than most of the mesoscutum. Mesoscutum 1.3× wider than long. Mesoscutum and scutellum uniformly covered by dense, silvered-coloured pilosity. Scutellum almost completely smooth. Scutellum length/width at base 1.2×. Scutellar suture shallow and thin with 8–9 costulae some of them confluent. Posterior band of scutellum polished. Scutellar lateral face with polished area about 1/3 the face height. Mesopleura smooth and glabrous, except for a few punctures and setae on the margins; sternaulus marked by a shallow impression with transverse striae. Crenulated sulcus separating meso and metapleura. Metapleura smooth in basal half, apical half punctuated and with setae; metapleura with a crenulated, longitudinal sulcus running from lower margin near the metacoxa through the spiracle. Metapleural carina with lamella. Propodeum weakly punctuate, almost smooth; propodeal areola absent but there is a short, postero-median longitudinal band of rugosity (consisting of several short carinae radiating from nucha).

Metasoma. Mediotergite 1 parallel sided for over 3/4 of its length, then slightly narrowing towards apex where it is rounded at posterior end; basal width/apical width 1.8×; length/apical width 3.6×; mediotergite 1 essentially unscultured except postero-laterally near apical margin; with broad excavation medially over anterior half. Mediotergite 2 subtriangular but with lateral margins weakly defined; basal width/apical width 0.3×; length/apical width 0.5×; essentially smooth, with fine, longitudinal grooves sublaterally, delimiting a central, raised region that is more or less rectangular in shape. Mediotergite 3 1.2× longer than mediotergite 2. Mediotergite 3 and following unsculptured, polished and with sparse setae. Hypopygium evenly sclerotized, truncated and slightly longer than apical tergites. Ovipositor sheaths very short (visible part 1/10 the length of metatibia), the tip blunt and with very sparse, tiny setae (those setae much shorter than hypopygium pilosity).

Legs. Metatibial inner spur 1.5× the length of outer spur, and 0.7× the length of metatarsomere 1. Metafemur 3.6× as long as wide. Protarsus with Protapanteles-like spine. Tarsal claws basally with a large lobe that extends more than half the claw length.

Wings. Fore wing vein R1a as long as stigma length; length of R1a about 5.0× as long as the distance between its end and the end of 3RSb. Vein r 0.8× the maximum width of stigma. Vein r meeting 2RS in a distinct angle marked by a knob. Vein 2M about the same length that vein (RS+M)b. Hindwing with margin of vannal lobe medially straight and without setae in the flat area.

Colour: Maxillary and labial palps, labrum, mandibles, scape, pedicel, tegula, wing base, all legs (except for metatibia apex which is darker), medio tergite 1 and most of sternite yellow. Flagellomere light brown; clypeus orange-brown. Mesosoma dark brown, except for most of propleura and pronotum, notauli, lateral margins and apical 1/4 of mesoscutum which are honey-orange. Head brownish-black. Rest of metasoma brown. Stigma and veins in forewing brown.

#### Distribution and biology.

This species represents the northernmost record of the genus. Nothing is known of its biology.

#### Comments.

Based on morphology only, the limits between Distatrix carolinae and Distatrix solanae seem weak; however, morphological similarities are common within this genus. For example: Distatrix solanae shares a number of characteristics with Neotropical species (see Grinter et al. 2009). I consider the Canadian specimen as a new and distinct species based on the major differences within the localities and habitats (California’s inner Coast Range and Oregon Cascade Mountains for Distatrix solanae; King Mountain in Gatineau Park, Quebec, for Distatrix carolinae). In the CNC there are several specimens representing at least another undescribed species from the Nearctic (southern and eastern US); the study of those specimen will clarify in time the limits of the North America species.

#### Etymology.

I dedicate this species to Caroline Boudreault (CNC), who likes so much to ski and enjoy the Gatineau Park. Your friendship, advices and jokes are always a great encouragement!

### Pseudapanteles gouleti

Fernández-Triana sp. n.

urn:lsid:zoobank.org:act:35425DE9-DD0B-4118-8851-617694A01EFA

**Figures 17–20.**
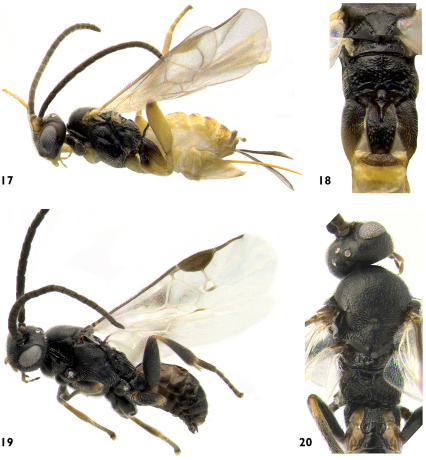
**17** Pseudapanteles gouleti, lateral **18** Pseudapanteles gouleti, mesosoma and mediotergites 1–3, dorsal **19** Venanus heberti, lateral **20** Venanus heberti, mesosoma and mediotergites 1–3 dorsal.

Figs 17–18 Supplementary Appendix 1 

#### Type locality.

Canada, Ontario, Ottawa, 45°21.365'N, 75°42.416'W.

#### Type material.

##### Holotype.

**Female** (CNC), with labels as follows: CANADA: ON, Ottawa, 45°21.365'N, 75°42.416'W, 13–23.vii.2007, H. Goulet, malaise trap, city garden; second label with Specimen ID: CAM 0253. CNC TYPE 23941.

##### Paratypes

(CNC): 1 ♀ and 6 ♂ same data than holotype except for collecting dates as follow: 13–23.vii.2007 (2 ♂), 30.vii-10.viii.2007 (3 ♂), 10.viii-1.ix.2007 (1 ♀, 1♂) [Specimens ID: CAM 0251, 0252, 0254–0258]; 1 ♀ Quebec, Hull, Malaise Trap, 10.viii.1965; 5 ♂ Quebec, Hull, Malaise Trap, 31.viii.1965; 2 ♀ Quebec, Old Chelsea, Summit King Mountain, 350 m, 22 and 27.viii.1965; 1 ♀ Ontario, Twp. Nepean, 25.viii.1949, H. A. Tripp col., reared from an immature case of Paraclemensia acerifoliella collected 10.v.1949; 1 ♀ Ontario, St. Lawrence Islands National Park, McDonald Island, 5.viii.1976; 4 ♂ Ontario, St. Lawrence Islands National Park, Thwartway Island, 1.viii.1976 (1 ♂), 2.viii.1976 (2 ♂), 12.ix.1976 (1 ♂); 1 ♀ Ontario, Innisville, 6.viii.1963, W. R. Mason.

#### Diagnosis.

Pseudapanteles gouleti is recognized by its more sculptured propodeum, with transverse carination all over its surface in addition to the median carina (the rest of the Nearctic species have the propodeum mostly smooth with only a median carina and at most a few, small transverse ridges radiating from base of median carina); the uniformly brown veins and stigma in the forewing (veins mostly hyaline and stigma hyaline centrally with margins light brown in the other species); mediotergite 1 fully sculptured, its basal 0.6 parallel-sided and then narrowing towards apex, its basal width about 1.2–1.3× its apical width (mediotergite 1 partially or fully smooth; barrel-shaped in Pseudapanteles nigrovariatus and Pseudapanteles sesiae or strongly narrowing from base to apex in Pseudapanteles dignus).

#### Description.

##### Female

Antenna length 2.2 mm (2.0–2.2 mm), slightly shorter than body length (2.6 mm, range: 2.2–2.7 mm) and forewing (2.7 mm, range: 2.3–2.7 mm). Head with glossa bilobate and long. Face with shallow and sparse punctures and sparse, uniformly distributed setae. Face width at antennal base/face width at clypeus edge: 1.2×; intertentorial pit distance/face width at clypeus edge: 0.5×; compound eye height/head height: 0.7×; head height/width: 0.9×; face width at antennal base/head maximum width: 0.6×; malar space/basal width of mandible 1.1×. Clypeus transversely narrow, its width/height: 4.5×. Length/width of flagellomeres: 1^st^ (2.3×), 2^nd^ (2.7×), 3^rd ^(2.3×), 8^th^ (2.0×), 14^th^ (1.0×), 15^th^ (1.0×), 16^th^ (1.0×). Ocelo-ocular distance/posterior ocelli diameter: 2.5×; distance betwen posterior ocelli/ocelli diameter: 1.6×.

Mesosoma. Pronotum XX. Mesoscutum uniformly sculptured by dense and well impressed punctures (distance between punctures about half their diameter). Mesoscutum 1.5× wider than long. Mesoscutum and scutellum uniformly covered by dense, silvered-coloured pilosity. Scutellum similarly sculptured than mesoscutum, though punctures slightly shallower and sparser. Scutellum length/width at base 1.2×. Scutellar suture thin and shallow, with 8–9 costulae. Posterior band of scutellum polished. Scutellar lateral face with polished area about 1/2 the face height. Except for a few punctures on the upper anterior margin, mesopleuron smooth and glabrous, setae over all of mesopleuron margins. Crenulated sulcus separating meso and metapleura. Metapleuron smooth in basal half, apical half punctate and with setae, metapleuron with a crenulated, longitudinal sulcus running from lower margin near metacoxa through spiracle. Metapleural carina with short lamella. Propodeum with median carina well defined and raised over its entire length; propodeum fully sculptured with transverse carinae, some radiating from the median carina.

Metasoma. Mediotergite 1 parallel sided for the basal 0.6× of its length, then narrowing towards apex, basal width/apical width 1.3× (1.2–1.3×); length/apical width 3.1×; mediotergite 1 with deep medial groove over its basal half, fully sculptured with longitudinal to transverse striae (except for a very small basal area surrounding the beginning of the groove and a small, polished apical knob). Mediotergite 2 transverse, subtriangular to trapezoidal in shape; basal width/apical width 0.4×; length/apical width 0.4×; fine, longitudinal striae covering most of the surface (sometimes apical third smooth). Mediotergite 3 more than twice the length of mediotergite 2. Mediotergite 3 and following unsculptured, polished and uniformly covered by sparse setae. Hypopygium striate, with acute tip protruding beyond apical tergites. Ovipositor sheaths fully setose, 1.0–1.2× as long as metatibia length.

Legs. Metatibial inner spur 1.4× the length of outer spur, and 0.47× the length of metatarsomere 1. Metafemur 3.2–3.5× as long as wide.

Wings. Vein R1a 1.2–1.3× as long as stigma length. Length of R1a about 6.0× as long as the distance between its end and the end of 3RSb. Vein r X the maximum width of stigma. Vein r and 2RS evenly curved to very slightly arched, with no clear limits between the two veins. Vein 2M about twice as long as vein (RS+M)b. Edge of vannal lobe of hindwing medially straight to slightly convex and with uniform length setae shorter than those at base and apex of lobe.

Colour: Labrum, mandibles (except for black tips), scape and pedicel yellow; maxillary and labial palps light yellow; clypeus orange-brown; rest of antenna and head brown. Mesosoma, basal half of metacoxa and mediotergite 1 dark brown to black; mediotergite 2 completely, mediotergite 3 and following centrally, apical half of metacoxae dorsally, metatarsi and apex of metatibia, light brown; tegula, rest of legs, tergites 3 and following laterally, and all sterna, yellow to light yellow almost white; stigma and veins in forewing brown.

##### Male

Males have mediotergite 3 and following almost completely brown, clypeus, scape and pedicel darker, and metacoxa fully brown. The flagellomeres are longer than those of females.

#### Variation.

Some specimens have lighter body coloration.

#### Molecular data.

Eleven specimens rendered full barcodes, with four haplotypes showing up to 0.3% of variation (1–2 bp). Those specimens were compared with one unauthenticated specimen of Pseudapanteles dignus, the only Nearctic species with data available in GenBank. Pseudapanteles gouleti is very distinctive, with more than 18% of base pairs different from the other species (Fig. 23).

#### Distribution and biology.

All specimens have been collected in an area bounded by the St Lawrence and Ottawa rivers (44º-46º N and 74º-75º W) near Canada’s capital. This is the northernmost known record of the genus Pseudapanteles. I studied 8 ♀ and 15 ♂ captured between mid July to mid September. One specimen was reared from the Maple Leafcutter, Paraclemensia acerifoliella (Fitch, 1856) (Incurvariidae). This is the third record of Braconidae parasitizing an incurvariid Lepidoptera; the other two being another Microgastrinae, Pholetesor ornigis (Weed, 1887), and a Braconinae, Bracon montowesei (Viereck, 1917); in all cases attacking the same incurvariid species (Marsh 1979; Yu et al. 2005; Whitfield 2006).

#### Etymology.

I dedicate this species to Henri Goulet (CNC) in whose backyard (a biodiversity gem in Ottawa, fondly called by CNC researchers as “Goulet National Park”) the holotype and several paratypes were collected. Henri wisely encouraged me to study the Microgastrinae and during four years has kindly given me access to his lab, collections and great expertise on many insect topics.

### Venanus heberti

Fernández-Triana sp. n.

urn:lsid:zoobank.org:act:B1DF493F-7D2D-46C4-B7E2-AC26D8EA06FE

Figs 19, 20 Supplementary Appendix 1

Venanus pinicola  Mason, 1981: 95 (in part). [Examined].

#### Type locality.

Canada, Prince Edward Island, Blooming Point, 46°24.486'N, 62°57.062'W.

#### Type material.

##### Holotype.

**Male** (CNC), with labels as follows: CANADA: PEI, Blooming Point, 46°24.486'N, 62°57.062'W, 23.vii.2008, fallow field, 6m, Goulet, Boudreault & Badiss, sweeping, #16. Second label with Specimen ID: MIC 000476. CNC TYPE 23942.

##### Paratypes

(CNC): 1 ♀ Annapolis Royal, NS, 7.ix.1945, J. McDunnough, ex Microlep. on Gaylussacia; 2 ♂ same data than holotype (Specimen ID: MIC 000474 and MIC 000475); 1 ♂ Bridgetown, NS, 2.ix.12, JES; 1♂ Sable Island, NS, 11–15.ix.1967, W.R.M. Mason; 1 ♂ Halifax, NS, 15.viii.1954, J. McDunnough, ex Caloptilia asplenifoliella; 1 ♂ Knowlton, QC, 19.viii.1929, G. S. Walley, ex larva on Myrica; 1 ♂ Kazabazua, QC, 19.viii.1933, G. S. Walley, ex larva on blueberry.

#### Diagnosis.

Venanus heberti is similar to Venanus pinicola Mason, 1981, and will run to that species in the recent key to the New World species (Whitfield et al. in press). Venanus pinicola is smaller (females: 1.6–1.9 mm, average=1.7 mm, N=8; males 1.7–2.4 mm, average=2.0 mm, N=5) than Venanus heberti (female: 2.2 mm; males: 2.0–2.4 mm, average=2.2, N=8). The size (width and height) of the second submarginal cell in the fore wing (compared to the length of vein r, the width and the length of stigma) is smaller in Venanus pinicola -usually the values represent 0.6–0.8 of similar proportions for Venanus heberti. The males of Venanus pinicola have its veins mostly pigmented (as have the females of both species), contrasting with mostly unpigmented veins in males of Venanus heberti. The two species have different geographical distributions: Venanus pinicola in west Canada/US (AB, BC, YT and ID) and Venanus heberti in Eastern Canada. The known hosts are different: the Gelechids Coleotechnites milleri (Busck, 1914) and Coleotechnites starki (Freeman, 1957) for Venanus pinicola; and the Gracillarid Caloptilia asplenifoliella (Darlington, 1949) for Venanus heberti. The two species also differ in 12 base pairs of the barcode region (more details below under the sections **Molecular data**, **Distribution and biology** and **Comments**).

#### Description.

##### Male

Antenna length 2.4 mm (1.9–2.4 mm), body length 2.4 mm (2.0–2.4 mm), forewing 2.1 mm (2.0–2.2 mm). Head with glossa truncate and short. Face smooth, with shallow punctures (separation between punctures larger than punctures diameter) and sparse, uniformly distributed setae. Face width at antennal base/face width at clypeus edge: 1.1×; intertentorial pit distance/face width at clypeus edge: 0.5×; compound eye height/head height: 0.7×; head height/width: 0.7×; face width at antennal base/head maximum width: 0.6×; malar space/basal width of mandible 1.0×. Clypeus transverse, its width/height: 3.6×. Ocelo-ocular distance/posterior ocelli diameter: 2.0× (2.0–2.4×); distance betwen posterior ocelli/ocelli diameter: 2.0×.

Mesosoma. Pronotum very smooth and polished, laterally with only the ventral groove well defined. Mesoscutum mostly smooth, with shallow but close punctures (distance between punctures 0.5–0.7 its diameter), punctures a sparser centrally along the posterior margin. Mesoscutum 1.2× (1.1–1.2×) wider than long. Mesoscutum and scutellum covered by sparse, silvered-coloured pilosity (sparser in the scutellum). Scutellum mostly smooth, with a few, shallow, very sparse punctures. Scutellum length/width at base 1.0×. Scutellar suture width 1/7 scutellum length, with 16 costulae not very well defined. Posterior band of scutellum polished. Scutellar lateral face with the polished area semicircular, 0.3–0.4× the face height. Mesopleuron smooth and glabrous on most of its surface, with sparse setae and punctures (distance between punctures usually twice or more its diameter) only on the anterior, ventral and posterior margins. Deep sulcus, with costulae, separating meso and metapleura. Metapleuron setose and punctured along anterior and ventral margins; lower ¼ of metapleuron rugulose, and with a broad, crenulated sulcus running from lower margin through spiracle. Metapleural carina lamellate and with costulae. Propodeum mostly rugulose, especially on the apical third (which is concave and delimited from the rest of the propodeum by a vague transverse carina); an obscure longitudinal carinae running centrally from base of propodeum until it reaches the transverse carina; transverse carina intersected posteriorly by several longitudinal, arched ridges radiating from nucha.

Metasoma. Mediotergite 1 widened and rounded apically, with its widest part subapically; basal width/apical width 0.9×; length/apical width 1.5; mediotergite 1 rugulose, apical ¼ with longitudinal striation laterally and two pits at each side of a central, polished area (like a knob) that reaches the posterior margin of tergite. Mediotergite 2 trapezoidal in shape, centrally smooth and polished, laterally rugulose; basal width/apical width 0.6×; length/apical width 0.6×. Mediotergite 3 twice the length of mediotergite 2. Mediotergite 3 and following unsculptured, polished and with few, sparse setae mostly along the posterior margin of tergites.

Legs. Metatibial inner spur 1.2× the length of outer spur, and 0.6× the length of metatarsomere 1. Metafemur 2.7× as long as wide.

Wings. Forewing vein R1a 0.7× as long as stigma length; length of R1a 2.7× as long as the distance between its end and the end of 3RSb. Vein r 0.6× (0.6–0.7×) the maximum width of stigma. Second submarginal cell height about the same length (or slightly smaller or larger) than vein r length; vein 2M 3.0× as long as vein (RS+M)b and 0.25–0.33× the stigma length. Edge of vannal lobe of hindwing covex and uniformly setose.

Colour: Mostly black to dark brown; pro- and meso- tibiae and tarsi yellowish brown, as it is apical 0.2× of metatibia, metatibial spurs, maxillary and labial palps. Wings hyaline, with most of veins transparent or whitish, except for C+Sc+R, R1, and 2M can be partially or totally pigmented; stigma brwon.

##### Female

Similar to male but with antenna (~1.0 mm) much shorter than body length (2.2 mm) and fore wing (2.0 mm). Antenna with a single row of placodes. Length/width of flagellomeres: 1^st^ (1.6×), 2^nd^ (1.1×), 8^th^ (1.1×), 14^th^ (1.2×), 15^th^ (1.3×). Length of flagellomere 2/flagellomere 14: 1.2×. Fore wing with most veins slightly pigmented (light brown in colour), and with larger and taller second submarginall cell (length of vein 2M half the stigma length, vein 2M almost twice the length of vein r). Metafemur thicker, 2.1× as long as wide. Hypopygium not folded nor striate, with slightly pointed tip not protruding beyond apical tergites. Ovipositor sheaths barely exerted from hypopygium, 0.1× as long as metatibia length; with sparse and minute setae.

#### Molecular data.

Full barcodesof 3 specimens of Venanus heberti and one specimen of the related species Venanus pinicola were obtained and compared (Fig. 24). The molecular data showed 12 (1.86 %) base pairs of difference between the two species.

#### Distribution and biology.

The species is widely distributed in Eastern Canada (QC, NS, PE), where it has been realibly reared from Gracillaria asplenifoliella. In the CNC there is one specimen of Venanus heberti from BC with a label stating it was reared from Caloptilia invariabilis (Braun, 1927). This has to be a labelling mistake because Coleotechnites invariabilis is only known from Eastern Canada (NS, ON, QC) and US, but has never been recorded from western Nearctic (De Prins and De Prins 2010).

#### Comments.

When Mason (1981) described Venanus pinicola he mentioned some variations in the specimens from Eastern Canada compared to the West, but considered that as intraspecific variation. The consistent, though subtle, morphological and molecular differences; different geographical distribution and hosts provide sufficient evidence to consider them as distinct species. Because of the similarities between the two species, four former paratypes of Venanus pinicola (in the CNC) are here transferred as paratypes of Venanus heberti.

#### Etymology.

I dedicate this species, recognized after DNA barcoding provided a first clue, to Paul Hebert (University of Guelph), as an appreciation for his support; and also for allowing the gathering of thousand of Microgastrinae barcodes –which will hopefully contribute in a significant way to the taxonomy of such a difficult and diverse group.

**Figures 21–24.**
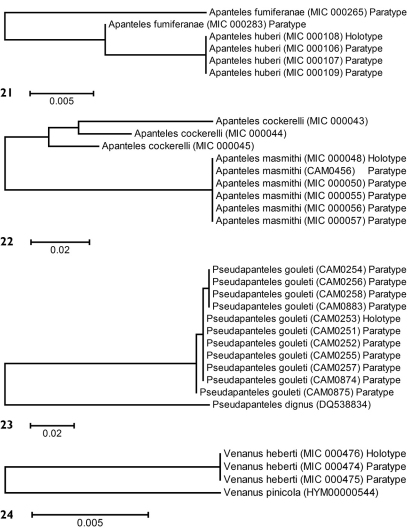
Neighbour-joining trees, K2P distance model. **21** Type material of Apanteles fumiferanae and Apanteles huberi **22** Type material of Apanteles masmithi and authenticated specimens of Apanteles cockerelli **23** Type material of Pseudapanteles gouleti and one unauthenticated specimen of Pseudapanteles dignus **24** Type material of Venanus heberti and one authenticated specimen of Venanus pinicola. Alphanumeric characters between parentheses refer to the specimens Sample ID (see Methods for more details). The number of specimens per species and its Sample IDs are detailed in the Supplementary Appendix 1.

## Checklist of genera and species of Microgastrinae for Canada and Alaska

### Genus Alphomelon Mason, 1981

This New World endemic genus was recently revised by Deans et al. (2003), but its distribution is mostly tropical and only one species is recorded from Canada. No additional species are expected from the region.

Alphomelon winniewertzae Deans, 2003. ON, QC. Also recorded from ENA and the NEO (Mesoamerica).

### Genus Apanteles Förster, 1862

This is one of the largest genera of Microgastrinae, with 35 species (32 of them described) recorded from the region. There are hundreds of unidentified specimens in the CNC and other collections, and the genus will have many more species when further studies can be carried out. Three species are left undescribed here until more studies of the Holarctic species allow establishing their identities with more accuracy. The only comprehensive key to the Nearctic species is in Muesebeck (1922), a relatively useful although outdated work. Mason (1974) and Fernández-Triana and Huber (2010) provided keys to species parasitizing tortricids -but those papers are missing many other species attacking a different spectrum of Lepidoptera. In his review of Apanteles sensu lato Papp (1976) and subsequent papers provided keys to the European species, and Chen and Song (2004) also provided a key to Chinese species. The genus badly needs a comprehensive review and, probably as importantly, a clarification of its current limits, a controversial and unsolved problem (e.g. Mason 1981; Whitfield 1995, 1997; van Achterberg 2002b).

Apanteles aristoteliae Viereck, 1912. NB, ON, QC. Distributed in the NEA.

Apanteles baldufi Muesebeck, 1968. ON. Also recorded from MI and MN in US.

Apanteles canarsiae Ashmead, 1898. ON, QC. Distributed in the ENA.

Apanteles carpatus (Say, 1836). BC, NB, ON. A cosmopolitan species.

Apanteles conanchetorum Viereck, 1917. NS, ON. Distributed in the ENA.

Apanteles corvinus Reinhard, 1880. NL. Distributed in the PAL, introduced in Canada (Raske, 1978) to control the birch casebearer moth Coleophora serratella (Coleophoridae).

Apanteles crassicornis (Provancher, 1886). AB, ON, SK, QC. Distributed mostly in the ENA, with some records on CNA and WNA.

Apanteles depressariae Muesebeck, 1931. NS, ON, QC. Distributed in the ENA.

Apanteles edwarsii Riley, 1889. ON, QC. Distributed in the ENA.

Apanteles ensiger (Say, 1836). MB, NS, ON, QC. Distributed in the ENA and CNA.

Apanteles epinotiae Viereck, 1912. ON. Distributed in the ENA and CNA.

Apanteles feltiae Viereck, 1912. SK. Distributed in the NEA.

Apanteles forbesi Viereck, 1910. MB, NS, ON. Distributed in the NEA.

Apanteles fumiferanae Viereck, 1912. AK, BC, MB, NB, NL, NT, ON, QC. Distributed in the NEA, with a record from Europe (Poland). Recent work done on this species has segregated several new species from Apanteles fumiferanae (e.g. Mason 1974; Fernández-Triana and Huber 2010; this study). However, morphology, barcoding, and host data strongly suggest that there are still more species under that name. An integrative approach will be needed to unravel this species-complex. In the meantime, the accuracy of current host records and “species” distribution should be taken with extreme caution. Solving this taxonomic mess will be very important because of the role played in the biological control of economically important pests, especially the tortricid genus Choristoneura.

Apanteles galleriae Wilkinson, 1932. BC. A cosmopolitan species.

Apanteles harti Viereck, 1910. ON. Distributed in the ENA.

Apanteles huberi Fernández-Triana, 2010 [present paper]. BC.

Apanteles jenniferae Fernández-Triana, 2010 [present paper]. NB, ON, QC.

Apanteles laricellae Mason, 1959. NB, ON, QC. Distributed in the ENA.

Apanteles masmithi Fernández-Triana, 2010 [present paper]. ON, NS.

Apanteles milleri Mason, 1974. BC, NB, NT, ON, QC. Distributed in the NEA (across Canada and northern US).

Apanteles morrisi Mason, 1974. BC, MB, NB, ON, QC. Distributed in the HOL (across Canada, northern US and Poland).

Apanteles nephoptericis (Packard, 1864). ON. Distributed in the NEA.

Apanteles petrovae Walley, 1937. AB, BC, NB, NL, ON, QC, SK. Distributed in the HOL. This species has always been considered as belonging to Apanteles by North America authors since its description (e.g. Mason 1974, 1981; Whitfield 1995); however, Papp (1988) transferred it to Dolichogenidea in his treatment of the European species, and that has been accepted by other workers. The generic identity of the species certainly seems controversial from both morphology and molecular data. For example, Nearctic specimens of Apanteles petrovae tend to cluster with Dolichogenidea instead of Apanteles (Fernández-Triana, unpublished data). Even from a detailed morphological redescription of the species (Mason 1974) it could be inferred that the species belongs to Dolichogenidea. However, I have carefully examined the holotype and found that the vannal lobe is medially flattened and with minute, sparse setae; a character that would put the species under the genus Apanteles. As stated before, our present understanding of those genera is far from complete; but pending more studies to clarify or improve the boundaries between them I prefer to keep the traditional treatment of the species as Apanteles.

Apanteles plesius Viereck, 1912. ON. Distributed in the ENA.

Apanteles polychrosidis Viereck, 1912. BC, MB, ON, QC. Distributed in the NEA.

Apanteles roughleyi Fernández-Triana, 2010 [present paper]. BC.

Apanteles samarshalli Fernández-Triana, 2010 [present paper]. ON. Also found in FL. See more comments of its distribution under the species description above.

Apanteles sodalis (Haliday, 1834). BC, NB, NL. Distributed in the HOL, introduced accidentally to Canada (Mason 1978).

Apanteles starki Mason, 1960. AB, BC. Distributed in WNA and China.

Apanteles victoriae Muesebeck, 1921. BC.

Apanteles xanthostigma (Haliday, 1834). NL. Distributed in the PAL and with two references from Uganda (Yu et al. 2005). Introduced in Canada (Williamson 1963), though there is no published data about its establishment.

Apanteles sp. 1 near *nephoptericis*. ON. Four specimens in CNC. Most likely it is a new species but, pending further study of the Holarctic fauna of Apanteles, it is not described in this paper.

Apanteles sp. 2 near *plesius*. QC. A recent paper (Fernández-Triana et al. 2009b) recorded three specimens from Frelishburg, QC, as a different but related species.

Apanteles sp. 3 near *pseudoglossae*. QC. A recent paper (Fernández-Triana et al. 2009b) recorded one specimen from Frelishburg, QC, as Apanteles pseudoglossae Muesebeck, 1921, which would represent a new record of the species for Canada. After checking the specimen and comparing it with other Nearctic material I now consider it a different species. Thus, the known distribution of Apanteles pseudoglossae at present does not extend to Canada –as stated by those authors- but it is restricted to IL, MD, MI and MN in the US.

**Figure 25.**
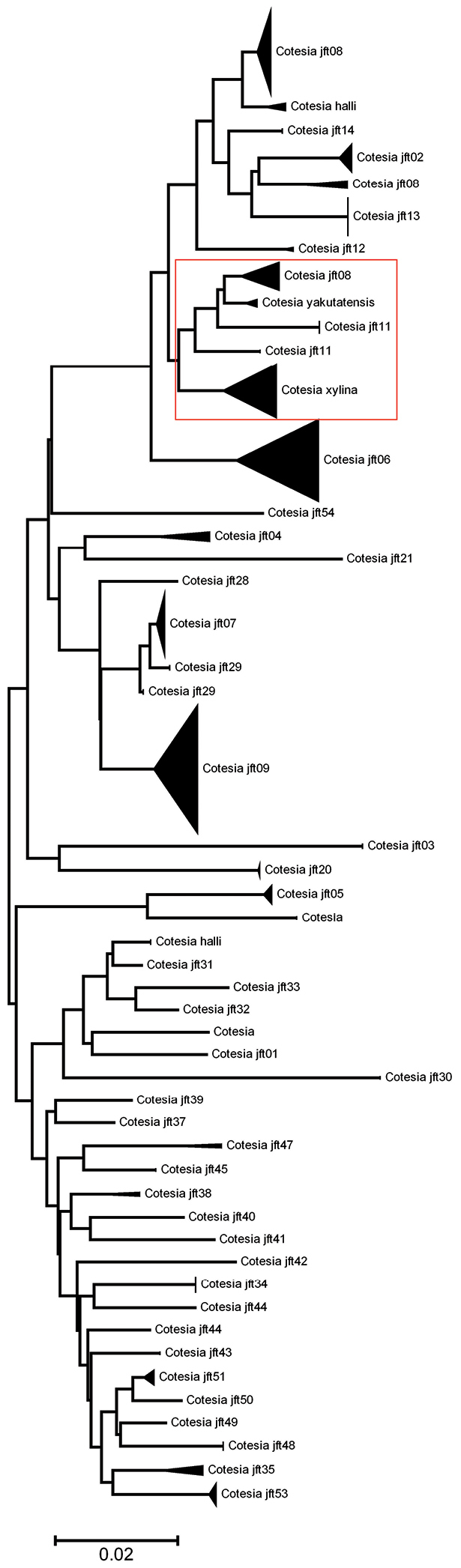
Neighbour-joining tree, K2P distance model, for Cotesia spp. from Canada and Alaska. The tree is cut in two sections to allow its display in a single page. The red square shows the complex of species related to Cotesia xylina and Cotesia yakutatensis (see explanation in the text, Checklist section). The number of specimens per species and its Sample IDs are detailed in the Supplementary Appendix 2.

### Genus Choeras Mason, 1981

There are keys to some Palearctic species (e.g. Tobias 1986; van Achterberg 2002a; Chen and Song 2004; Kotenko 2007), although the genus has never been properly revised. Three species are known from the Nearctic region (Whitfield 1995) and all of them occur in Canada. Additionally, in the CNC there are many specimens from western Canada that most likely represent several species; but lacking comprehensive studies of the genus I am taking the conservative approachof keeping them as belonging to one species for now.

Choeras consimilis (Viereck, 1911). MB, NB, ON, QC. Distributed in the HOL.

Choeras insignis (Muesebeck, 1938). BC. Also recorded from CA in the US.

Choeras tiro (Reinhard, 1880). NL, NS, PE. Distributed in the HOL

Choeras sp. AB, BC.

### Genus Clarkinella Mason, 1981

There are only two described species from this genus, one of them from Canada (Mason 1981). I do not expect more species from the region.

Clarkinella canadensis Mason, 1981. ON. Known previously only from holotype, two additional specimens were recently collected in Ottawa.

### Genus Cotesia Cameron, 1891

This is probably the largest genus in the region. It is also one of the most cohesive taxa within Microgastrinae (Mason 1981), although sometimes specimens of Protapanteles (see below on remarks of that genus) could be confused with Cotesia. The keys in Muesebeck (1922) and Papp (1990) will only work partially for identifying specimens, because a plethora of new taxa remain undescribed. Here 55 species are recorded for the region studied (51 of them described). At least four undescribed species are also mentioned but lack of a comprehensive study on the Holarctic fauna prevents me to formally describe them here. Additionally, thousands of specimens in collections remain unidentified; some of them likely represent many additional species.

Cotesia acauda (Provancher, 1886). NS, ON, QC. Distributed in the ENA.

Cotesia acronyctae (Riley, 1871). AB, ON, SK. Distributed in the NEA.

Cotesia anisotae (Muesebeck, 1921). NB, ON. Distributed in the NEA.

Cotesia atalantae (Packard, 1881). AB, MB, ON, SK, QC. Distributed in the NEA.

Cotesia autographae (Muesebeck, 1921). NL, MB, QC. Distributed in the NEA.

Cotesia brevicornis (Wesmael, 1837). AB. Distributed in the HOL.

Cotesia carduicola (Packard, 1881). ON. Distributed in the NEA.

Cotesia cerurae (Muesebeck, 1926) ON. QC. Distributed in the ENA.

Cotesia cingiliae (Muesebeck, 1931). AB, BC, NB, NS, ON, QC. Distributed in the ENA.

Cotesia clisiocampae (Ashmead, 1903). ON. Previously known from north-eastern US. First record to Canada.

Cotesia congestiformis (Viereck, 1923). AK.

Cotesia congregata (Say, 1836). MB, NB, ON, PE. Distributed in the NEA and the NEO.

Cotesia crambi (Weed, 1887). QC. Distributed in the ENA.

Cotesia cyaniridis (Riley, 1889). ON, QC. Distributed in the NEA.

Cotesia diacrisiae (Gahan, 1917). ON, QC. Distributed in the NEA.

Cotesia diversa (Muesebeck & Walkley, 1951). MB. Previously known only from Connecticut, first record to Canada.

Cotesia electrae (Viereck, 1912). BC. Distributed in the NEA and Mexico.

Cotesia enypiae (Mason, 1959). BC.

Cotesia fiskei (Viereck, 1910). AB, BC, MB, NB, NL, NS, ON, SK. Distributed in the US, first record to Canada.

Cotesia flaviconchae (Riley, 1881). ON. Distributed in the US, first record to Canada.

Cotesia flavicornis (Riley, 1889). MB, ON. Distributed in the US, first record to Canada.

Cotesia glomerata (Linnaeus, 1758). BC, NB, ON, QC. A cosmopolitan species.

Cotesia griffini (Viereck, 1911). AB, NB, QC. Distributed in the NEA.

Cotesia halisidotae (Muesebeck, 1931). BC, MB, ON, QC. Distributed in the NEA.

Cotesia hallii (Packard, 1877). NT, NU. Also recorded from Greenland.

Cotesia hemileucae (Riley, 1881). NB. Distributed in the US, first record to Canada.

Cotesia hyphantriae (Riley, 1887). BC, MB, NB, NS, ON, QC. Distributed in the HOL and Mexico.

Cotesia koebelei (Riley, 1889). BC. Distributed in the US, first record to Canada.

Cotesia laeviceps (Ashmead, 1890). AB, BC, MB, NB, ON, QC, SK. Distributed in the NEA.

Cotesia limenitidis (Riley, 1871), NS, ON. Distributed in the NEA.

Cotesia lunata (Packard, 1881). QC. Distributed in the US, first record to Canada.

Cotesia lyciae (Muesebeck, 1938). QC. Previously known only from Maine, first record to Canada.

Cotesia mahoniae (Mason, 1975). BC. Distributed in the WNA.

Cotesia melanoscela (Ratzeburg, 1844). BC, NB, NL, NS, ON, PE, QC. Distributed in the HOL.

Cotesia murtfeldtae (Ashmead, 1898). MB, ON, QC. Distributed in the NEA.

Cotesia nemoriae (Ashmead, 1898). MB, NL, NS, ON, QC, SK. Distributed in the NEA.

Cotesia olenidis (Muesebeck, 1922). BC.

Cotesia parastichtidis (Muesebeck, 1921). BC, NB, NS, ON. Distributed in the NEA.

Cotesia phobetri (Rohwer, 1915). AB, NL, ON. Distributed in the NEA.

Cotesia plathypenae (Muesebeck, 1921). BC, MB. Distributed in the NEA.

Cotesia pyraustae (Viereck, 1912). ON. Distributed in the ENA.

Cotesia pyrophilae (Muesebeck, 1926). ON. Distributed in the ENA.

Cotesia rubecula (Marshall, 1885). BC, ON, QC. A cosmopolitan species.

Cotesia rufocoxalis (Riley, 1881). NS. Distributed in the ENA and CNA.

Cotesia schizurae (Ashmead, 1898). ON. Distributed in the US, first record to Canada.

Cotesia scitula (Riley, 1881). NS, ON. Distributed in the ENA and CNA.

Cotesia smerinthi (Riley, 1881). BC, ON, QC. Distributed in the NEA.

Cotesia teleae (Muesebeck, 1926). AB, BC. Distributed in the US, first record to Canada. The new distribution towards western NEA is significant.

Cotesia tmetocerae (Muesebeck, 1921). NS.

Cotesia xylina (Say, 1836). AB, MB, NS, ON, QC. Distributed in the NEA. Whether this species is valid or not has been questioned by Muesebeck (1921) and Papp (1986) who considered it a synonym of Cotesia yakutatensis or Cotesia tibialis (Curtis) respectively. Some of the Canadian specimens seem closely related to those of Cotesia yakutatensis. However, when barcoded specimens from Canadian Cotesia xylina, Cotesia yakutatensis and related specimens are analyzed, 5 distinct clusters are obtained (Fig. 25; Supplementary Appendix 2). Based on the keys of Muesebeck (1921), (Papp (1986, 1987, 1989) and van Achterberg (2006), some of those specimens might be Cotesia halli, Cotesia melanoscela, Cotesia eliniae, Cotesia tetricus (the last two not yet recorded from the Nearctic), or just new species. The only way to solve this species-complex would be a study of the genus at Holarctic level, which is beyond this paper.

Cotesia yakutatensis (Ashmead, 1902). AK, BC, MB, QC. Distributed in the NEA. See comments under Cotesia xylina.

Cotesia sp. 1. MB. This species is treated as Cotesia jft01 in a paper currently under review (Fernández-Triana et al. unpublished). The only available specimen, a male from Burnt Site, Churchill, MB, runs to Cotesia nemoriae in Muesebeck (1921) and to Cotesia subordinanius in Papp (1986), but it is neither of those species. Its very large metacoxae (half the length of the metasoma) are very distinctive and seem to support its status as a new species.

Cotesia sp. 2. MB, NL, NU, PE, QC, YT. This species is treated as Cotesia jft09 in a paper currently under review (Fernández-Triana et al. unpublished). Additional specimens, mostly from northern localities, have been found later in several Canadian provinces and territories. It is related to Holarctic species with short antennae —e.g. Cotesia arctica (Thompson), Cotesia astrarches (Marshall, 1889), and Cotesia tenebrosa (Wesmael) —but differs from all of the species keyed by Papp (1976). Most likely it is a new species but, pending further study of the Holarctic fauna, it is not described in this paper.

Cotesia sp. 3. AB, MB, ON, SK, YT. The specimens grouped here (in the CNC) comprise those near Cotesia xylina and/or Cotesia yakutatensis that are still unresolved but are clearly different species (Fig. 25; see also comments under Cotesia xylina).I am taking the conservative approachof considering all those specimens as belonging to one species for now –though they most likely represent several.

Cotesia sp 4. ON. Over 30 specimens reared from Plutella xylostella in Ottawa. It is none of the known species of Cotesia parasitizing Plutella, and most likely represents a new species. It is not described here because of the same reason than previous species.

**Figure 26.**
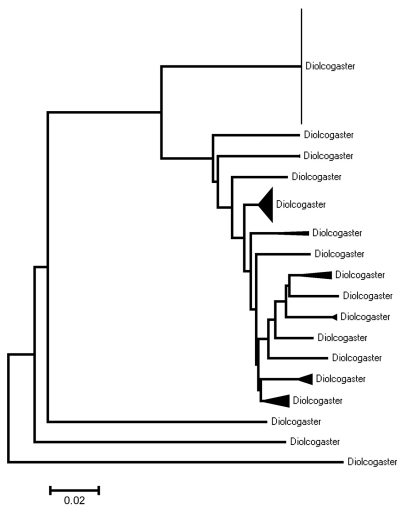
Neighbour-joining tree, K2P distance model, for Diolcogaster spp. from Canada and Alaska. The species are not named pending an upcoming review of the genus (Choi WY and Whitfield JB, unpublished). The number of specimens per species and its Sample IDs is detailed in the Supplementary Appendix 2.

### Genus Deuterixys Mason, 1981

This is a small genus and the Nearctic species were revised by Whitfield (1985). No additional species are expected to be represented in the region.

Deuterixys pacifica Whitfield, 1985. BC.

### Genus Diolcogaster Ashmead, 1900

At least 10 species (7 of them described) are recorded here; however, it is clear that the actual number of species is much higher. For example: based only on the specimens of Diolcogaster with barcode sequences currently available (135 specimens, 122 of them with more than 500 bp) there are more than 17 clearly delimited species in the region, even if a very conservative approach is taken (Fig. 26; Supplementary Appendix 2). No further efforts are made here to deal with those specimens because there is a pending taxonomic review at Nearctic level (Choi WY and Whitfield JB, unpublished) that should improve our present understanding of the genus.

Diolcogaster auripes (Provancher, 1886) NB, ON, QC. Distributed in the ENA and CNA.

Diolcogaster bakeri (Muesebeck, 1922). ON, QC, SK. Distributed in the NEA and NEO. Recently recorded in Canada (QC) by Fernández-Triana et al. (2009b).

Diolcogaster brevicauda (Provancher, 1886). QC. Distributed in the ENA.

Diolcogaster facetosa (Weed, 1888). AB, BC, ON, QC. Distributed in the NEA and China.

Diolcogaster garmani (Ashmead, 1900). ON. Distributed in the US, first record to Canada.

Diolcogaster schizurae (Muesebeck, 1922). BC, ON. Distributed in the NEA.

Diolcogaster scotica (Marshall, 1885). BC, QC. Distributed in the HOL.

Diolcogaster sp. 1. MB. This species is treated as Diolcogaster jft01 in a paper currently under review (Fernández-Triana et al. unpublished). It does not key to any described species, and the barcoding data suggests it is one of the most distinctive and unique species within the genus.

Diolcogaster sp 2. AK. This is the first record of the genus for Alaska. The specimen, collected on July, 1959 in Umiat (69°22'N, 152°09’W) and housed in the CNC is very distinctive and also represents the northernmost known record of the genus.

Diolcogaster sp. 3. NT. One specimen, almost as far north as the previous species, was collected on July, 1971 at Kovaluk River (69°11'N, 131°00’W) and housed in the CNC. However, it represents a different species.

### Genus Distatrix Mason, 1981

This is a predominantly tropical genus, and a revision of the New World species was published recently (Grinter et al. 2009). I have found a new species reaching as north as QC, Canada, and representing the northernmost known record.

Distatrix carolinae Fernández-Triana, 2010 [present paper]. QC.

### Genus Dolichogenidea Viereck, 1911

The keys dealing with Apanteles (mentioned above in the treatment of that genus) also cover the species of Dolichogenidea. Both genera are easily confused (Mason 1981; Whitfield et al. 2009), and the correct generic assignment of some of the species is often limited by subtle and subjective characters. A comprehensive study of those genera (plus a few others, see for example, van Achterberg 2002b) is badly needed to redefine its limits with accuracy. In the meantime, 19 species of Dolichogenidea (17 of them described) are recorded here for the region studied.

Dolichogenidea absona Muesebeck, 1965. AB, BC, NB, NL, NS, MB, ON, PE, QC. Distributed in the NEA.

Dolichogenidea breviventris (Ratzeburg, 1848). Previously recorded as Dolichogenidea mesoxantha (Ruschka, 1971) by Whitfield (1995) in his list of Nearctic species. However, Dolichogenidea mesoxantha was synomized under Dolichogenidea breviventris by Papp (1978). NL. Distributed in the HOL.

Dolichogenidea cacoeciae (Riley, 1881). ON, QC. Distributed in the NEA.

Dolichogenidea californica (Muesebeck, 1921). AB, BC, ON, QC. Distributed in the WNA, here recorded for the first time from ENA.

Dolichogenidea clavata (Provancher, 1881). ON, QC. Distributed in the NEA.

Dolichogenidea coleophorae (Wilkinson, 1938). NL. Distributed in the PAL, introduced in Canada. In the CNC there are specimens reared in NL from the birch casebearer moth Coleophora serratella (Coleophoridae). This species was transferred to Apanteles by van Achterberg (2002b) but I am keeping it as Dolichogenidea based on its vannal lobe medially convex and uniformly covered by setae of the same length.

Dolichogenidea homoeosomae (Muesebeck, 1933). SK. Distributed in the NEA and Cuba.

Dolichogenidea lacteicolor (Viereck, 1911). NB, NS, QC. Distributed in the HOL and also some records in the Oriental region. This species was transferred to Apanteles by van Achterberg (2002b) but I am keeping it as Dolichogenidea based on its vannal lobe medially convex and uniformly covered by setae of the same length.

Dolichogenidea laspeyresiae (Viereck, 1913). BC. Distributed in the WNA.

Dolichogenidea longicauda Wesmael (1837). BC. Distributed in the HOL. Recently Fernández-Triana and Huber (2010) commented on the changing generic status of this species within the last years, and considered it as belonging to Dolichogenidea, a decision that is followed here.

Dolichogenidea melanopa (Viereck, 1917). BC, PE. Previously known from Connecticut. First record to Canada.

Dolichogenidea paralechiae (Muesebeck, 1932). NB, ON, QC. Distributed in the NEA.

Dolichogenidea phthorimaeae (Muesebeck, 1921). ON. Distributed in the NEA and Honduras.

Dolichogenidea renaulti Mason, 1974. NB, NS, ON, QC. Distributed in the ENA.

Dolichogenidea solenobiae (Walley, 1935). ON, QC. Distributed in the ENA.

Dolichogenidea thujae (Muesebeck, 1935). ON, QC. Distributed in the ENA.

Dolichogenidea tischeriae (Viereck, 1912). QC. Distributed in the NEA. A recent paper (Fernández-Triana et al. 2009b) recorded the species for the first time in Canada, but erroneously mentioned it as Apanteles tischeriae. The species was transferred to Dolichogenidea by Mason (1981).

Dolichogenidea sp. 1 near *cacoeciae*. BC. Twelve specimens in the CNC were considered by Mason as a different species. Pending further study of the Holarctic fauna of Dolichogenidea, it is not described in this paper.

Dolichogenidea sp. 2. ON. Considered by Mason as a new species belonging to the Dolichogenidea laevigata species-group. The CNC has 14 specimens that were reared from two Tortricidaehosts: Proteotera aesculana (4 specimens) and Argyroploce albiciliana (10 specimens). Pending further study of the Holarctic fauna of Dolichogenidea, it is not described in this paper.

### Genus Exix Mason, 1981

This genus has one Nearctic species (Mason 1981), known from only one specimen in Canada.

Exix columbica Mason, 1981. BC.

### Genus Exoryza Mason, 1981

Like the previous genus, only one species is known from the Nearctic (Mason 1981), with no more species seen in collections (Whitfield 1995).

Exoryza minnesota Mason, 1981. ON. Distributed in the ENA.

### Genus Glyptapanteles Ashmead, 1904

This genus is considered one of the most diverse and dominant genera in tropical regions (e.g. Whitfield 1995; Whitfield et al. 2009), but is still very commonly seen and specious in Alaska/Canada, reaching even to the tip of Ellesmere Island (+82°N). Papers covering Apanteles (mentioned above under that genus) will deal with some species, but the limits of the genus are controversial (e.g. van Achterberg 2002b; and discussion under Protapanteles below) and there are no updated keys to the species. Many unidentified specimens remain in collections and the recorded species here (10, nine of them described) are just a fraction of the actual number.

Glyptapanteles alticola (Ashmead, 1902). AK, BC, NB. Distributed in the NEA. From the material housed in the CNC, Papp’s (1983) statement that Glyptapanteles alticola is not different from Glyptapanteles fulvipes (Haliday) seems valid. However, both species are kept here as valid until the type material can be studied.

Glyptapanteles compressiventris (Muesebeck, 1921). MB, NT, NU, QC. Distributed in the HOL.

Glyptapanteles flavovariatus (Muesebeck, 1921). BC, ON. Distributed in the NEA. First record to Canada.

Glyptapanteles fulvipes (Haliday, 1834). AB, NT, NU, QC. Distributed in the HOL. First record to Canada.

Glyptapanteles militaris (Walsh, 1861). MB, NB, ON, QC. A cosmopolitan species.

Glyptapanteles pallipes (Reinhard, 1880). AK, BC, NB, ON, QC. A cosmopolitan species. I am including under this species also Glyptapanteles longicornis (Provancher, 1886), a name mentioned as a valid species by Whitfield (1995) in his list of Nearctic species. However, Glyptapanteles longicornis was synonymized under Glyptapanteles pallipes by Papp (1988), an arrangement that has been accepted by subsequent authors (e.g. Yu et al. 2005; Kotenko 2007).

Glyptapanteles sarrothripae (Weed, 1887). BC, NS, ON. Distributed in the ENA, here recorded for the first time for WNA.

Glyptapanteles websteri (Muesebeck, 1921). AB, NB, QC. Distributed in the ENA.

Glyptapanteles sp. 1 near *alticola*. MB. Specimens from Manitoba form around half a dozen of distinct clusters based in barcoding data that might well represent different species related to Glyptapanteles alticola (Fernández-Triana et al. unpublished data). The barcoding differences are also supported by slight morphological differences (e.g. antennae colour, relative length of the last flagellomeres, puncture density of head, seta density and length on the mesoscutum, scutellum punctures, wing base colour, propodeal carination, hind leg colour —especially tibia and tarsi— sculpture of mediotergites 1 and 2). However, without having molecular data from the type material and/or a comprehensive taxonomical review of the genus within the Holarctic, an unequivocal assignment of specimens is not possible at present. I am taking the conservative approachof considering all specimens as one species for now –though they probably represent many species.

### Genus Hygroplitis Thomson, 1895

No more species are expected to be found within the Nearctic region (Whitfield 1995).

Hygroplitis melligaster (Provancher, 1886). MB, NB, NS, ON, QC. Distributed in the ENA, here recorded for the first time from CNA.

### Genus Hypomicrogaster Ashmead, 1898

Some specimens in the CNC seem to be different species than the two described species here recorded for the region. There is currently a review of the genus underway for the New World fauna (Valerio A, pers. com.) and thus no further attempt is made here to deal with those unidentified specimens.

Hypomicrogaster ecdytolophae (Muesebeck, 1922). NS, ON, QC. Distributed in the NEA and the NEO.

Hypomicrogaster zonaria (Say, 1836). NB, NS, ON, QC. Distributed in the NEA

Hypomicrogaster sp. ON. This is a new species that will be described elsewhere (Valerio A, pers. com.).

### Genus Iconella Mason, 1981

This genus has never been revised, though key to some Palearctic species can be found in Chen and Song (2004) and Kotenko (2007). There is only one described species within the Nearctic (from US and the Neotropics) but none was previously recorded from Canada. I have found at least two species in Canada but, pending a study of the Holarctic fauna, the species are not described here.

Iconella sp. 1. NB. In the CNC.

Iconella sp. 2. ON, BC. In the CNC.

### Genus Illidops Mason, 1981

This genus has never been revised, though Kotenko (2007) provided a key to the Russian Far East. There is only one described species within the Nearctic (from southern US) and a few undescribed have been mentioned from Arctic Canada and the Rocky Mountains (Mason 1981). Some of the Canadian species lack the posterior medioapically desclerotized tergites and/or the convergent eyes that characterize the genus. The majority of the specimens available were collected more than 30 years ago, and only one recent specimen rendered a full barcode, but for 18 older specimen partial barcodes (120–292 bp) were obtained. Both morphology studies and barcoding indicate that, even under the most conservative approach, there are at least four species in the studied region. Pending a larger study of the Holarctic fauna, the species are not described here.

Illidops sp. 1. MB. This species is treated as Illidops jft01 in a paper currently under review (Fernández-Triana et al. unpublished).

Illidops sp. 2. MB. This species is treated as Illidops jft02 in a paper currently under review (Fernández-Triana et al. unpublished).

Illidops sp. 3. MB. This species is treated as Illidops jft03 in a paper currently under review (Fernández-Triana et al. unpublished).

Illidops sp. 4. AB, MB, NS, NT, ON, QC. Additional material from the provinces mentioned here is housed in the CNC. They are different from the previous three species, and probably represent more than one species, but for now are kept provisionally as one species.

### Genus Lathrapanteles Williams, 1985

This genus was described and its species revised by Williams (1985) and no new additions are expected. The validity and relationships of this genus to other Microgastrinae might be questioned when future studies are made.

Lathrapanteles fuscus Williams, 1985. BC, MB, NS, NT, QC. Distributed in the NEA.

Lathrapanteles heleios Williams, 1985. ON.

Lathrapanteles papaipemae (Muesebeck, 1921). NL, ON, QC. Distributed in the NEA.

### Genus Microgaster Latreille, 1804

Muesebeck (1920), Nixon (1968) and Papp (1984) provided keys covering all known species of the region, and Whitfield (1995) estimated that only a handful of species were likely to be added to the Nearctic though he also remarked the need for a full appraisal of the North American fauna. Here I consider 13 species for Canada/Alaska, two of them needing further study to clarify their specific status.

Microgaster brittoni Viereck, 1917. ON. Distributed in the ENA.

Microgaster canadensis Muesebeck, 1922. AB, BC, MB, NB, NS, ON, PE, QC, SK. Distributed in the NEA.

Microgaster congregatiformis Viereck, 1917. AB, MB, ON. Distributed in the NEA.

Microgaster deductor Nixon, 1968. MB. Distributed in the PAL, recorded from Canada in a paper currently under review (Fernández-Triana et al. unpublished).

Microgaster epagoges Gahan, 1917. BC, ON, QC. Distributed in the NEA.

Microgaster gelechiae Riley, 1869. ON, QC. Distributed in the NEA.

Microgaster hospes Marshall, 1885. ON, QC. Distributed in the HOL.

Microgaster leechi Walley, 1935. BC, MB, ON, QC. Distributed in the NEA.

Microgaster pantographae Muesebeck, 1922. ON. Distributed in the HOL.

Microgaster peroneae Walley, 1935. AK, BC, NB, NL, NS, ON, QC. Distributed in the NEA.

Microgaster messoria Haliday, 1834. ON, QC. Distributed in the HOL, was introduced in the NEA at the beginning of the XX century.

Microgaster sp. 1. MB. This species is treated as Microgaster jft01 in a paper currently under review (Fernández-Triana et al. unpublished). The only specimen available, a male, appears related to Microgaster sticticus Ruthe, 1858 (from the Palearctic region) but more material is needed before its status can be clearly defined.

Microgaster sp. 2. MB. This species is treated as Microgaster jft02 in a paper currently under review (Fernández-Triana et al. unpublished). The small size (about 2.5 mm), eyes subparallel, mesoscutum rugulose and small length of ovipositor make this species related to the European Microgaster fischeri Papp, 1960, but it is most likely a new species.

### Genus Microplitis Förster, 1862

This is a diverse genus in the Holartic, and there are no satisfactory keys to species available. Whitfield (1995) estimated that more than half of the Nearctic species were undescribed. Indeed, there are hundreds of specimens in collections that likely represent many new species. Here I report 21 species (19 of them described), but this is just a fraction of the actual number. Barcoding data available for 681 specimens with more than 500 bp (Supplementary Appendix 3) reveals almost 60 species, even under the most conservative approaches.

Microplitis alaskensis Ashmead, 1902. AK, AB, BC, MB, NS, ON, QC. Distributed in the NEA.

Microplitis autographae Muesebeck, 1922. AB, ON. Distributed in the CNA.

Microplitis bradleyi Muesebeck, 1922. AB, BC. Distributed in the WNA.

Microplitis carteri Walley, 1932. AB.

Microplitis ceratomiae Riley, 1881. NB, NS, ON, QC, SK. Distributed in the NEA.

Microplitis confusus Muesebeck, 1922. NB, ON. Distributed in the NEA.

Microplitis crenulatus (Provancher, 1888). QC. Distributed in the ENA.

Microplitis gortynae Riley, 1881. ON. Distributed in the NEA.

Microplitis hyphantriae Ashmead, 1898. AB, ON, QC. Distributed in the NEA.

Microplitis impressus (Wesmael, 1837). MB, ON, QC. Distributed in the HOL.

Microplitis kewleyi Muesebeck, 1922. AB, MB, NB, ON, QC. Distributed in the NEA.

Microplitis laticinctus Muesebeck, 1922. QC. Distributed in the ENA.

Microplitis mamestrae Weed, 1887. BC. Distributed in the NEA.

Microplitis maturus Weed, 1888. BC, ON, QC. Distributed in the ENA and CNA.

Microplitis melianae Viereck, 1911. AB, ON. Distributed in the NEA.

Microplitis plutellae Muesebeck, 1922. ON, QC, SK. Distributed in the HOL.

Microplitis quadridentatus (Provancher, 1886). ON. Distributed in the ENA.

Microplitis scutellatus Muesebeck, 1922. AB. Distributed in the NEA.

Microplitis varicolor Viereck, 1917. MB, NB, ON, QC. Distributed in the NEA.

Microplitis sp. 1. MB. This species is treated as Microplitis jft01 in a paper currently under review (Fernández-Triana et al. unpublished). Without study of authenticated material from Europe is difficult to conclude, but according to the descriptions provided by Papp (1984) and van Achterberg (2006) this species is closely related to Microplitis coactus (Lundbeck, 1896), which was previously known just from Greenland and Iceland. The specimens from Churchill may represent a different and new species, with larger metafemur.

Microplitis sp. 2 near *varicolor*. MB. There are numerous specimens in the CNC that are related to Microplitis varicolor but seem different species -based on both barcoding and morphological differences. A comprehensive study of Microplitis at least at Nearctic level will be needed before those specimens can be assigned to species.

### Genus Paroplitis Mason, 1981

There is only one known Nearctic species and no more are expected (Mason 1981; Whitfield 1995).

Paroplitis beringianus Mason, 1981. AK, BC.

### Genus Pholetesor Mason, 1981

The Nearctic species were revised by Whitfield (2006) and the genus is reasonably covered. However, Palearctic species need to be dealt with altogether with the Nearctic ones to avoid duplication of descriptions. For that reason, I am treating two of the 20 species recorded here as undescribed for now.

Pholetesor bedelliae (Viereck, 1911). BC, NS, ON, QC. A cosmopolitan species.

Pholetesor caloptiliae Whitfield, 2006. ON. Distributed in the ENA.

Pholetesor circumpscriptus (Nees, 1834). AK. A cosmopolitan species.

Pholetesor glacialis (Ashmead, 1902). AK, BC.

Pholetesor longicoxis Whitfield, 2006. QC. Distributed in the ENA.

Pholetesor masneri Mason, 1981. ON. Distributed in the ENA.

Pholetesor masoni Whitfield, 2006. AB, BC, NS, ON, QC. Distributed in the NEA and Mexico.

Pholetesor ornigis (Weed, 1887). MB, NB, NS, ON. QC. Distributed in the NEA.

Pholetesor pedias (Nixon, 1973). ON. Van Achterberg (1997) synonymised this species under Pholetesor exiguus (Haliday, 1837) but a latter comprehensive review of Nearctic Pholetesor (Whitfield 2006) kept the Pholetesor pedias name. Distributed in the HOL.

Pholetesor pinifoliellae Whitfield, 2006. ON, QC. Distributed in the NEA.

Pholetesor rhygoplitoides Whitfield, 2006. ON, QC. Distributed in the NEA.

Pholetesor rohweri (Muesebeck, 1921). NB, ON. Distributed in the ENA.

Pholetesor salalicus (Mason, 1959). BC. Distributed in the HOL.

Pholetesor salicifoliellae (Mason, 1959). BC, MB, NB, NS, ON, QC. Distributed in the NEA.

Pholetesor thuiellae Whitfield, 2006. NB, ON, QC. Distributed in the ENA.

Pholetesor variabilis Whitfield, 2006. AB, BC, ON, SK. Distributed in the NEA.

Pholetesor viminetorum (Wesmael, 1837). AB, AK, BC, MB, NS, YT. Distributed in the HOL.

Pholetesor zelleriae Whitfield, 2006. MB, ON, QC. Distributed in the NEA.

Pholetesor sp. 1. MB. This species is treated as Pholetesor jft01 in a paper currently under review (Fernández-Triana et al. unpublished). This is likely a new species related to Pholetesor powelli, Pholetesor bedelliae and Pholetesor thuiellae but clearly different from them. A study within the context of Holarctic species is badly needed.

Pholetesor sp. 2. MB. This species is treated as Pholetesor jft02 in a paper currently under review (Fernández-Triana et al. unpublished). Two male specimens (Sample ID: 07PROBE-22417, 07PROBE-23399) differ slightly from Pholetesor viminetorum regarding veins r and 2RS, length of metatibial spurs and shape of tergite 1 and 2. The barcode variation between these two species was 1.94%, and there are also two character states differences within the D2 region of the nuclear gene 28S. The combination of these three lines of evidence suggests that those males are a separate species from Pholetesor viminetorum. However, pending a study of the Holarctic fauna, the species is not described here.

### Genus Protapanteles Ashmead, 1898

Altogether with Apanteles, the limits of this genus are one of the most controversial (e.g. Mason 1981; Whitfield 1995, 1997; van Achterberg 2002b; Yu et al. 2005). Mason (1981) provided some characters that supposedly defined the genera, but even within a geographical restricted area such as Canada, there are considerable variation (e.g. specimens with propodeum sculptured like a typical Cotesia, instead of smooth; mediotergite 1 strongly narrowing toward apex like typical Glyptapanteles; specimens looking like Sathon; etc). The North American workers have usually considered it to be a rather small genus, and have kept the other genera separated, but I am not sure if that is the best arrangement, or at least Glyptapanteles and Sathon should be part of an expanded Protapanteles genus -similar to the proposal of van Achterberg (2002b). Solving those problems is beyond the scope of this paper and for now I am following Whitfield’s (1995) arrangement of the Nearctic species.

Protapanteles alaskensis (Ashmead, 1902). AK, BC, MB, NL. Distributed in the NEA.

Protapanteles paleacritae (Riley, 1881). BC, MB, NL, NS, ON. Distributed in the NEA.

Protapanteles phigaliae (Muesebeck, 1919). NB, ON. Distributed in the NEA.

Protapanteles phlyctaeniae (Muesebeck, 1929). ON. Distributed in the ENA and CNA.

Protapanteles sp. 1. AB, BC, SK, MB. A significant number of specimens from western Canada is included here, most of them from reared material housed in the CNC and the Northern Forestry Centre, Edmonton. I am taking the conservative approachof considering all specimens as one species for now –though they likely represent several species

### Genus Protomicroplitis Ashmead, 1898

This small genus had been reported within the Nearctic from central and eastern US, as north as NY (Yu et al. 2005). In the CNC there are several specimens of Pholetesor calliptera captured near Ottawa (Metcalfe and Stittsville) which represent the first record of the genus for Canada and the northernmost distribution in North America. No more species are expected to be found in the region.

Protomicroplitis calliptera (Say, 1836). ON. Distributed in the NEA.

### Genus Pseudapanteles Ashmead, 1898

This New World genus is mostly found in the tropics, with a few species reaching to the US. Here I record two species (one of them new) for Canada, expanding further north the known distribution of the genus.

Pseudapanteles gouleti Fernández-Triana, 2010 [present paper]. ON, QC.

Pseudapanteles sesiae (Viereck, 1912). ON. Two specimens from Niagara Falls represent the northernmost record of the species. Distributed in the NEA.

### Genus Rasivalva Mason, 1981

All the Nearctic species of this genus are dealt with in Muesebeck (1922), and Whitfield (1995) stated that no clearly undescribed species had been seen in collections. I report here an additional undescribed Canadian species that does not fit within the described ones.

Rasivalva perplexa (Muesebeck, 1922). BC, NB, ON. Distributed in the NEA.

Rasivalva rugosa (Muesebeck, 1922). ON, QC. Distributed in the NEA. New record to Canada.

Rasivalva stigmatica (Muesebeck, 1922). AB, BC, QC. Distributed in the NEA. New record to Canada.

Rasivalva sp. ON, NB. Two female specimens in the CNC that are different to the previous species but that are not dealt with further until a study of the Holarctic fauna is done.

### Genus Sathon Mason, 1981

The limits of this genus are controversial (see above comments under Protapanteles and also Whitfield et al. 2002, 2009). Williams (1985) provided a key to species.

Sathon cinctiformis (Viereck, 1911). ON, QC. Distributed in the ENA.

Sathon masoni (Williams, 1988). AK, NU, NT. Distributed in the NEA.

Sathon neomexicanus (Muesebeck, 1921). AB, AK, BC, MB, NL, NT, ON, PE. Distributed in the NEA.

Sathon papilionae (Williams, 1988). AK, BC.

### Genus Venanides Mason, 1981

A small genus with four described species, one of them from the Nearctic (Mason 1981); with an additional couple of undescribed ones in southern US (Whitfield 1995). It is not likely that more species will be found in Canada/Alaska.

Venanides xeste (Mason, 1981). MB, ON. Distributed in the NEA and Brazil.

### Genus Venanus Mason, 1981

The genus was recently revised by Whitfield et al. (in press), but from the seven recognized species within the New World, only one was recorded from the Nearctic. The barcoding data revealed a new species that had been overlooked by Mason (1981); it is clear now that the specimens from eastern and western Canada are different (see more comments on the two species in the section describing the new species).

Venanus pinicola (Mason, 1981). AB, BC, YT. Distributed in the WNA.

Venanus heberti Fernández-Triana, 2010 [present paper]. NS, QC, PE.

## Supplementary Material

XML Treatment for Apanteles huberi

XML Treatment for Apanteles jenniferae

XML Treatment for Apanteles masmithi

XML Treatment for Apanteles roughleyi

XML Treatment for Apanteles samarshalli

XML Treatment for Distatrix carolinae

XML Treatment for Pseudapanteles gouleti

XML Treatment for Venanus heberti

## Supplementary Material

Appendix I. Comprehensive data for type material of Microgastrinae described in this paper where DNA barcodes were obtained. The Sample ID and Process ID fields allow to retrieve all molecular and collection information for every specimen through BOLD (http://www.barcodinglife.org). File format: Microsoft Excel (1997–2003). doi: 10.3897/zookeys.63.565-app.I

Appendix II. Comprehensive data for all specimens of genera Cotesia and Diolcogaster where DNA barcodes were obtained. The Sample ID and Process ID fields allow to retrieve all molecular and collection information for every specimen through BOLD (http://www.barcodinglife.org). File format: Microsoft Excel (1997–2003). doi: 10.3897/zookeys.63.565-app.II

Appendix III. Comprehensive data for all specimens of genus Microplitis where DNA barcodes were obtained. The Sample ID and Process ID fields allow to retrieve all molecular and collection information for every specimen through BOLD (http://www.barcodinglife.org). File format: Microsoft Excel (1997–2003). doi: 10.3897/zookeys.63.565-app.III
